# Supplementary material for: Brief intervention to reduce fatigue impact in patients with inflammatory arthritis: design and outcomes of a single-arm feasibility study
Source: BMJ Open. 2022 Jul 18;12(7):e054627. doi: 10.1136/bmjopen-2021-054627 (PMC9297231; doi:10.1136/bmjopen-2021-054627)
Supplement: Supplementary data [file bmjopen-2021-054627supp001.pdf]

Fatigue: Reducing its Effects through individualized support Episodes in Inflammatory Arthritis (FREE-IA)

Summary of the intervention

The intervention is designed to provide rheumatology health professionals with a tool kit to support patients with an inflammatory arthritis (IA) to self-manage their fatigue impact. There are two parts to the intervention: health professional training and the intervention manual.

Training covers:
- Cognitive behavioural approaches (CBA)
- Experiences from a fatigue clinic and validating fatigue
- Identifying individual fatigue drivers and starting patient engagement
- Daily activity diaries
- Socratic or ‘guided discovery’ questions
- Boom and bust or withdrawal and avoidance
- SMART goal setting and practice with clinical vignettes
- Discussions of sleep, stress and the meaning of acceptance in IA

The manual provides the materials to deliver up to four sessions. Sessions 1 & 2 are core and Sessions 3 & 4 are optional.

- Session 1: Engagement and validation (establish that fatigue is an issue that the patient wishes to address with the health professional and engage them in taking some responsibility/action).
- Session 2: Review daily diary, goals, and action planning (review the patient’s daily diary and reflect on whether this fits a boom/bust pattern of activity or a withdrawal and avoidance pattern of activity).
- Session 3: Sleep and rest (discuss potential factors contributing to disrupted or poor-quality sleep, forming helpful habits, and strategies to help the patient reduce their worry about sleep).
- Session 4: Stress and relaxation (support the patient to make connections between stressful circumstances, their fatigue, and how they cope with it, including the patient identifying their own symptoms of stress and recognising that stress comes from the interaction between circumstances (what’s happening) and their response (thoughts and feelings and behaviours) to the circumstances).

At the end of each session, there is a checklist. At the back of the manual, there are session handouts for patients and tutor materials. There is homework and patients are asked to complete a daily activity diary between sessions and to try out changes to their activity and routine that they have discussed with the health professional.

The intervention is based on a cognitive-behavioural approach (CBA) using an ‘Ask, don’t tell’ approach. CBA is based on the idea that symptoms do not shape how a patient responds to their IA, but rather the meaning that they make of those symptoms (e.g., that fatigue is unfair or a disaster). For this reason, health professionals see patients in clinic with similar symptoms/levels of disease, yet different ways of coping and different outcomes. To unpick this response, the patient needs to identify the links between their thoughts, feelings, and behaviours, and how these might be driving or exacerbating their fatigue. The health professional can help the patient to work out these links by asking questions to prompt the patient to reflect, including alternative ways of doing things. This can help the patient to generate their own ideas for ways forward and help ensure that the patient focuses on what is important to them. These are then translated into concrete goals for the patient to try.
