# Supplementary material for: Brief intervention to reduce fatigue impact in patients with inflammatory arthritis: design and outcomes of a single-arm feasibility study
Source: BMJ Open. 2022 Jul 18;12(7):e054627. doi: 10.1136/bmjopen-2021-054627 (PMC9297231; doi:10.1136/bmjopen-2021-054627)
Supplement: Supplementary data [file bmjopen-2021-054627supp002.pdf]

SUPPLEMENTARY TABLE S1 (PERCENTAGE OF COMPLETE/IMPUTED RESPONSES)

Measure	Time Point 0	Time Point 1	Time Point 2
BRAF-NRS Fatigue Effect (0-10)	100%	87.00%	84.78%
BRAF-NRS Coping (0-10)	89.13%	73.91%	73.91%
RAID Final Score (0-10)	89.13%	73.91%	73.91%
BRAF-MDQ Physical Severity (0-22)	89.13%	73.91%	73.91%
BRAF MDQ Living with Fatigue (0-21)	89.13%	73.91%	73.91%
BRAF-MDQ Cognitive (0-15)	89.13%	73.91%	73.91%
BRAF-MDQ Emotional (0-12)	89.13%	73.91%	73.91%
BRAF-MDQ Total (0-70)	89.13%	73.91%	73.91%
MHAQ Mean Score (0-4)	89.13%	71.74%	73.91%
HCCQ (1-7)	84.78%	73.91%	78.26%
RASE (28-140)	82.61%	71.74%	76.09%
