# Supplementary material for: Brief intervention to reduce fatigue impact in patients with inflammatory arthritis: design and outcomes of a single-arm feasibility study
Source: BMJ Open. 2022 Jul 18;12(7):e054627. doi: 10.1136/bmjopen-2021-054627 (PMC9297231; doi:10.1136/bmjopen-2021-054627)
Supplement: Author's manuscript [file bmjopen-2021-054627.draft_revisions.pdf]

BMJ Open is committed to open peer review. As part of this commitment we make the peer review history of every article we publish publicly available.

When an article is published we post the peer reviewers' comments and the authors' responses online. We also post the versions of the paper that were used during peer review. These are the versions that the peer review comments apply to.

The versions of the paper that follow are the versions that were submitted during the peer review process. They are not the versions of record or the final published versions. They should not be cited or distributed as the published version of this manuscript.

BMJ Open is an open access journal and the full, final, typeset and author-corrected version of record of the manuscript is available on our site with no access controls, subscription charges or pay-per-view fees (http://bmjopen.bmj.com).

If you have any questions on BMJ Open's open peer review process please email info.bmjopen@bmj.com

BMJ Open

A brief intervention to reduce fatigue impact in patients with inflammatory arthritis: design and outcomes of a single-arm feasibility study

Journal:	*BMJ Open*
Manuscript ID	bmjopen-2021-054627
Article Type:	Original research
Date Submitted by the Author:	04-Jul-2021
Complete List of Authors:	Dures, Emma; Bristol Royal Infirmary, Academic Rheumatology; University of the West of England<br>Bridgewater, Susan; University of the West of England<br>Abbott, Bryan ; Bristol Royal Infirmary<br>Adams, Jo; University of Southampton<br>Berry, Alice; University of the West of England<br>McCracken, Lance; Uppsala University<br>Creanor, Siobhan; University of Exeter<br>Hewlett, Sarah; University of the West of England<br>Lomax, Joe; Plymouth University<br>Ndosi, Mwidimi; Bristol Royal Infirmary, Academic Rheumatology Unit; University of the West of England, Nursing and Midwifery<br>Thorn, Joanna; University of Bristol<br>Urban, Marie; Bristol Royal Infirmary<br>Ewings, Paul; Taunton and Somerset NHS Foundation Trust
Keywords:	MEDICAL EDUCATION & TRAINING, REHABILITATION MEDICINE, RHEUMATOLOGY

SCHOLARONE™ Manuscripts

I, the Submitting Author has the right to grant and does grant on behalf of all authors of the Work (as defined in the below author licence), an exclusive licence and/or a non-exclusive licence for contributions from authors who are: i) UK Crown employees; ii) where BMJ has agreed a CC-BY licence shall apply, and/or iii) in accordance with the terms applicable for US Federal Government officers or employees acting as part of their official duties; on a worldwide, perpetual, irrevocable, royalty-free basis to BMJ Publishing Group Ltd ("BMJ") its licensees and where the relevant Journal is co-owned by BMJ to the co-owners of the Journal, to publish the Work in this journal and any other BMJ products and to exploit all rights, as set out in our licence.

The Submitting Author accepts and understands that any supply made under these terms is made by BMJ to the Submitting Author unless you are acting as an employee on behalf of your employer or a postgraduate student of an affiliated institution which is paying any applicable article publishing charge ("APC") for Open Access articles. Where the Submitting Author wishes to make the Work available on an Open Access basis (and intends to pay the relevant APC), the terms of reuse of such Open Access shall be governed by a Creative Commons licence – details of these licences and which Creative Commons licence will apply to this Work are set out in our licence referred to above.

Other than as permitted in any relevant BMJ Author's Self Archiving Policies, I confirm this Work has not been accepted for publication elsewhere, is not being considered for publication elsewhere and does not duplicate material already published. I confirm all authors consent to publication of this Work and authorise the granting of this licence.

For peer review only

A brief intervention to reduce fatigue impact in patients with inflammatory arthritis: design and outcomes of a single-arm feasibility study

Emma Dures[1,2], Susan Bridgewater[1,2], Bryan Abbott[2], Jo Adams[3], Alice Berry[1,2], Lance M McCracken[4], Siobhan Creanor[5], Sarah Hewlett[2], Joe Lomax[6], Mwidimi Ndosi[1,2], Joanna C Thorn[7], Marie Urban[2], Paul Ewings[8]

Author affiliations

[1]Academic Rheumatology, University Hospitals Bristol and Weston NHS Foundation Trust, Bristol, UK

[2]Faculty of Health and Applied Sciences, University of the West of England, Bristol, UK

[3]Health Sciences, University of Southampton, Southampton, UK

[4]Department of Psychology, Uppsala University, Uppsala, Sweden

[5]Exeter Clinical Trials Unit, College of Medicine & Health, University of Exeter, UK

[6]Medical Statistics Group, Faculty of Health, University of Plymouth, Plymouth, UK

[7]Bristol Medical School, University of Bristol, Bristol, UK

[8]Department of Research, Somerset NHS Foundation Trust, Taunton, UK

Corresponding author

Emma Dures

Academic Rheumatology

The Bristol Royal Infirmary

Bristol BS2 8HW

UK

emma2.dures@uwe.ac.uk

For peer review only

ABSTRACT

Objectives

Patients with inflammatory arthritis report that fatigue is challenging to manage. We developed a manualised, one-to-one, cognitive-behavioural intervention, delivered by rheumatology health professionals (RHPs). FREE-IA (Fatigue - Reducing its Effects through individualised support Episodes in Inflammatory Arthritis) tested the feasibility of RHP training, study design, intervention delivery, and outcome collection, ahead of a potential trial of clinical and cost-effectiveness.

Methods

In this single-arm feasibility study, eligible patients were ≥18 years, had a clinician-confirmed diagnosis of inflammatory arthritis and scored ≥6/10 on the BRAF NRS Fatigue Effect. Following training, RHPs delivered 2–4 sessions to participants. Baseline data were collected before the first session (T0), and outcomes at six weeks (T1) and six months (T2). The proposed primary outcome was fatigue impact (BRAF NRS Fatigue Effect). Secondary outcomes included fatigue severity and coping, disease impact and disability, and measures of therapeutic mechanism (self-efficacy and confidence to manage health).

Results

Eight RHPs at five hospitals delivered 113 sessions to 46 participants. Of a potential 138 primary and secondary outcome responses at T0, T1 and T2, there were 13 (9.4%) and 27 (19.6%) missing primary and secondary outcome responses, respectively. Results indicated improvements in all measures except disability, at either T1 or T2, or both.

Conclusions

This study showed it was feasible to deliver the intervention, including training RHPs, and recruit and follow-up participants with high retention. While there was no control group, within-group improvements were observed, providing evidence of promise of the intervention and support for moving towards a definitive trial.

For peer review only - http://bmjopen.bmj.com/site/about/guidelines.xhtml

Strengths and limitations of this study

- This study has established that rheumatology health professionals can train and deliver a brief, low-cost intervention for fatigue in inflammatory arthritis.
- The low levels of attrition and high levels of data completeness suggest the outcomes collected are appropriate for a definitive trial.
- The within-group improvements that were observed provide evidence of promise for the intervention.
- The lack of a control arm means that the feasibility/acceptability of randomisation has not been tested, and the improvements in outcomes could have arisen from regression to the mean or the small sample size.

For peer review only

INTRODUCTION

Inflammatory arthritis (IA) is a group of multi-systemic, auto-immune conditions characterised by pain, joint swelling and stiffness, and fatigue. The most common of these conditions is rheumatoid arthritis (RA).[1] Around 400,000 adults in the United Kingdom (UK) have RA and approximately three quarters of people are of working age when they are diagnosed.[2] Challenges for patients with IA include unpredictable fluctuations in symptoms, functional disability, and managing complex medication regimens.[3] Treatment options include pharmacological, non-pharmacological, and surgical interventions to control symptoms, prevent joint damage and improve mobility and function.[4] In the UK, treatment is typically provided in secondary care by multi-disciplinary rheumatology health professionals (RHPs), including physicians, nurse specialists, occupational therapists, and physiotherapists.

Fatigue is a common and distressing symptom in IA.[5] An international study of >6,000 patients found that one out of every two was severely fatigued, defined as scoring ≤35 on the SF-36 Vitality Scale.[6] Despite the high prevalence and impact of the symptom, patients perceive that often their fatigue is not addressed in rheumatology consultations.[7] UK research with >1,200 patients found that 82% wanted support to manage the impact of pain and fatigue.[8] RHPs have reported that they recognise that fatigue is an issue for patients but there is a lack of evidence-based resources that they can use in clinical practice.[9]

Fatigue in IA is associated with inflammation, pain, disability, sleep, depression and health beliefs, implying complex, multi-causal pathways.[10] A systematic review found that biologic treatments in patients with active RA can lead to a small to moderate improvement in their fatigue, suggesting that optimal disease activity management should be part of fatigue management.[11] However, biologic treatments are not prescribed for IA-related fatigue and there is evidence that patients can experience fatigue during remission.[12] A systematic review for non-pharmacological interventions concluded that physical activity and

For peer review only - http://bmjopen.bmj.com/site/about/guidelines.xhtml

psychosocial interventions, including cognitive-behavioural therapy (CBT), provide benefit in relation to self-reported fatigue in adults with RA.[13] This evidence has underpinned several CBT-based self-management interventions for fatigue.[14, 15] Although clinically effective they are highly structured, stand-alone interventions comprising at least six patient contact sessions. Consequently, they are time-consuming for patients to attend and for RHPs to deliver.

In response, we designed a brief, one-to-one intervention that aims to reduce fatigue impact by supporting patients to identify the thoughts, feelings and behaviours perpetuating their fatigue. Patients then use this understanding as the basis for making adaptive behaviour changes and enhancing their coping skills. The intervention is based on self-determination theory, which addresses motivation and competence to behave in effective and healthy ways; self-efficacy, a belief in one's ability to successfully engage in a course of action; and guided discovery (the 'Ask don't tell' approach rather than didactic information and advice-giving).[16-18] The intervention was designed by a multi-disciplinary team from nursing (SH), occupational therapy (JA) and psychology (LM, ED) and written as a manual. It comprises 2-4 sessions, each designed to last 20-30 minutes (Table 1). The first two sessions are core and designed to take place face-to-face and within two weeks. Up to two additional optional sessions can take place face-to-face or remotely, for example by telephone or video, within the subsequent four weeks.

TABLE 1: OVERVIEW OF INTERVENTION STRUCTURE AND CONTENT

Our study design was informed by the Medical Research Council's framework for developing and evaluating complex interventions.[19] Before investing in a definitive randomised controlled trial (RCT) to test an intervention's clinical and cost effectiveness (evaluation stage), the research team should have a reasonable expectation that the intervention could have a worthwhile effect, based on existing evidence and theory (development stage). They

For peer review only - http://bmjopen.bmj.com/site/about/guidelines.xhtml

should also examine whether the evaluation procedures are likely to be deliverable and acceptable (feasibility stage). Researchers are advised to use a mix of quantitative and qualitative methods to resolve the main uncertainties that might impede study delivery. To achieve this, we designed the feasibility study FREE-IA (Fatigue - Reducing its Effects through individualised support Episodes in Inflammatory Arthritis). Our aims were to:

- design and deliver intervention training to RHPs;
- recruit patients to the intervention;
- determine the completeness of outcome measurement data collection from patients who participated in intervention sessions;
- and identify the optimum approach for a cost-effectiveness evaluation to be conducted alongside a definitive RCT.

We also examined the acceptability of the intervention from the perspectives of patients who participated and RHPs who undertook training and delivery, via telephone interviews. These data are reported separately.

ETHICS APPROVAL STATEMENT

Ethics approvals for the study were granted by the South West - Frenchay Research Ethics (REC ref. 15/SW/0207). All participants provided written informed consent prior to taking part in the study.

MATERIALS AND METHODS

We used a single-arm feasibility study design comprising three phases:

- Phase 1: delivery of intervention training to RHPs
- Phase 2: patient recruitment and intervention delivery
- Phase 3: data collection and analysis

For peer review only - http://bmjopen.bmj.com/site/about/guidelines.xhtml

Phase I: we developed and delivered intervention training face-to-face. We included overviews of the IA fatigue evidence-base, underpinning psychological theories, and materials from the manual (cognitive components); skills demonstrations from the training team (modelling/illustrational component); skills practice using rheumatology-specific vignettes, with observation and feedback from the training team (experiential/behavioural component); and a problem-based learning approach, with RHPs using examples from their clinical practice.[20] Training was designed and delivered by ED, SH, LM and patient research partners MU and BA.

Phase II: individual secondary care sites made local decisions about their optimum strategy to invite patients to participate in the study. Eligibility criteria were rheumatology patients at a participating site; age 18 years and over with a clinician-confirmed diagnosis of IA; with a score ≥6/10 on the BRAF NRS Fatigue Effect[21] and with fatigue that they considered recurrent, frequent, and/or persistent; and who were not accessing support for their fatigue at the time of invitation. Patients who were unable to complete questionnaires in English unaided and/or patients lacking capacity to give informed consent were not eligible. Patients interested in participating completed and mailed their screening sheet to the study coordinator SB, who assessed their eligibility for the study. Following confirmation of eligibility, SB mailed a baseline data pack to patients who were interested in taking part. The pack comprised a consent form, and a questionnaire to collect demographic and clinical data and the proposed outcome measures to be used in the definitive RCT (see phase III). SB asked patients to complete the baseline data pack, including the consent form, and to bring it to their first intervention session.

After training, RHPs delivered intervention sessions to recruited patients. To inform patterns of uptake, amendments to the intervention and the cost of delivery, we asked RHPs to record the number and duration of intervention sessions delivered to each participant and the mode of delivery, for example face-to-face, by telephone or by video. Once they had

For peer review only - http://bmjopen.bmj.com/site/about/guidelines.xhtml

experience of delivery, we asked RHPs to audio-record the intervention sessions, if the participant consented, to assess how the intervention was delivered. We designed a pro-forma to guide assessment of competence and fidelity to the intervention. It comprised two parts: (i) inclusion of intervention content/topics and (ii) use of facilitative approaches by the RHP. In each section, research fellow AB scored the extent to which content was present and made notes to include examples and reflections. This information was for process evaluation purposes and not as feedback for the RHPs delivering the intervention.

Phase III: after baseline (T0), we collected quantitative outcomes data from participants at two time-points: six weeks post-intervention (T1) and six months post-intervention (T2). We defined post-intervention as six weeks after core session 1 because it covered the maximum intended period of exposure to the intervention. Our likely primary outcome in a future RCT is fatigue impact, measured using the BRAF-NRS Fatigue Effect.[21] We also collected secondary outcomes:

- BRAF-NRS Fatigue Severity[21]
- BRAF-NRS Fatigue Coping[21]
- Rheumatoid Arthritis Impact of Disease (RAID)[22]
- BRAF Multi-dimensional Questionnaire (BRAF-MDQ)[21]
- Modified Health Assessment Questionnaire (MHAQ)[23]

Measures of therapeutic mechanism:

- The Rheumatoid Arthritis Self-Efficacy Scale (RASE)[24]
- The Perceived Health Competence Scale (PHCS)[25]
- The Health Care Climate Questionnaire (HCCQ)[26]

SB collected the proposed primary outcome by telephone and the secondary outcomes via an outcome measures pack that was mailed to participants at T1 and T2. Participants were asked to complete the questionnaires and mail them back.

For peer review only - http://bmjopen.bmj.com/site/about/guidelines.xhtml

The FREE-IA Project Management Group approved analysis plans for the statistical outcomes and health economics. Methodologists PE, JL and SC conducted analysis of the statistical outcomes. For each self-reported questionnaire, the total scale and subscale scores were calculated in line with published guidance, including the use of imputation for unanswered questions (Supplementary Table 1). Outcome scores are reported as means and standard deviations, plus ranges, at each of the three time points. In addition, the mean change from T0 to T2 for each (sub)scale, with 95% confidence intervals, is presented.

Health economic outcomes were analysed by health economist JT. Health-related quality of life (EQ-5D-5L)[27] was collected at T0, T1 and T2, and valued using the van Hout crosswalk method based on UK population preferences.[28] Mean quality-adjusted life years (QALYs) were calculated over the six months of follow-up. A bespoke resource-use questionnaire was developed in consultation with patient partners, covering (1) NHS & personal social services (PSS) and (2) patient perspectives. An estimate of the cost of delivering the intervention itself was derived from study records. Standard sources were used to assign unit costs (2019) to each of the resources measured [29-32] and mean usage (e.g., appointments), mean costs and standard deviations were calculated over the six months of follow-up using all available cases. A non-comparative cost–consequences matrix was constructed.

PATIENT AND PUBLIC INVOLVEMENT

The research study, including the question, was developed with patient research partners Bryan Abbott (BA) and Marie Urban (MU), who have experience of living with inflammatory arthritis and fatigue. BA and MU were co-applicants in the funding application and are co-authors on this manuscript. The study was also discussed with the Patient Advisory Group in the Rheumatology Department of the Bristol Royal Infirmary. BA and MU reviewed all patient-facing literature, shaped the bespoke health economics questionnaire, supported

For peer review only - http://bmjopen.bmj.com/site/about/guidelines.xhtml

delivery of the intervention training, provided additional materials for RHPs delivering the intervention, advised on recruitment and helped to interpret the study findings. After study completion, they reviewed the written summaries that were sent to study participants, including patients and RHPs who had taken part.

ANALYSIS AND RESULTS

Delivery of intervention training to RHPs

We delivered face-to-face training three times, with different RHPs each time. In total, 12 RHPs (eight nurses, two occupational therapists, one associate rheumatology practitioner, and one clinical research practitioner) from six hospitals attended. The first training took place over two days at the hospital where the central study team are based, with seven RHPs from four sites and lasted for approx. 13 hours. Subsequently, one site withdrew from the study after their two RHPs had attended training but before recruiting patients, due to logistical challenges of intervention delivery at their hospital. Subsequently, two new sites joined the study, with training delivered over one and a half days (approx.10 hours) at the same central study team hospital to four RHPs. The third training lasted for one day (approx. five hours) and was delivered by ED at the hospital of an individual RHP from one of the new sites who had been unable to attend the group session with colleagues.

Patient recruitment and intervention delivery

A total of 46 patients were recruited to the FREE-IA study (Figure 1, Table 2). The overall recruitment rate was 0.22 participants per hospital per month, however, most sites did not recruit continuously over the duration of the recruitment period. The conversion rate, based on the number of participants recruited divided by the number screened, was 52.1% (63/121). Six of the 63 patients (9.5%) who expressed interest in participating were ineligible and/or declined to participate. Of the remaining 57 patients, five did not provide consent (8.8%) and three declined an invitation to take part (5.3%). One site did not invite an eligible patient because they had reached their target recruitment and one site stopped recruitment

For peer review only - http://bmjopen.bmj.com/site/about/guidelines.xhtml

early due to COVID-19, with the local team unable to invite two interested and eligible patients to participate in the study. This left 46 patients who provided written consent and who provided a proposed primary outcome at baseline.

FIGURE 1: FLOW DIAGRAM

Eight RHPs delivered 113 intervention sessions across five sites and duration ranged from 10-120 minutes (mean 44 minutes). One RHP took consent but did not deliver the intervention. At two sites, all intervention sessions were delivered by one RHP. At the three other sites, the number of intervention sessions delivered by each RHP varied. Of the total 46 participants, 39 (84.8%) completed the two core sessions. Seven (15.2%) attended one session, 16 (34.8%) attended two sessions, 18 (39.1%) attended three sessions, and five (10.9%) attended the maximum four possible sessions. Mode of delivery was face-to-face, except for four optional sessions, which were delivered by telephone. Session 2 of the intervention was delivered within the desired two-week timeframe for 37% of the participants who attended at least the two core sessions, with a mean of 21 days between sessions.

Twenty-five intervention sessions were audio-recorded across three sites; two sites did not record any sessions. AB evaluated all the audio-recordings and SB and ED analysed a sub-set independently. There was a high level of agreement between the team members in relation to the audio-recordings that were analysed in triplicate. The main insights were that:

- Most RHPs followed the manual in a linear way, but some adopted a more flexible approach guided by patients' fatigue-related support needs.
- RHPs used the materials to prompt discussion, initially to explore fatigue drivers and daily diaries, and later to explore goal setting, sleep, and stress.
- When it was difficult for patients to identify unhelpful behaviour patterns, some RHPs were more directive.

For peer review only - http://bmjopen.bmj.com/site/about/guidelines.xhtml

- Longer appointments allowed for linking thoughts and feelings with behaviours, developing goals, and exploring behaviour patterns.
- RHPs who had more time and/or experience and/or knew the patient from previous clinical appointments tended to explore negativity towards change with more confidence.

TABLE 2: FREE-IA PARTICIPANT DEMOGRAPHICS

For peer review only

Data completeness and summary of patient-reported outcome measures

There were 13 (9.4%) missing proposed primary outcome responses from 11 participants (T0 = 0, T1 = 6, T2 = 8) and 27 (19.6%) missing secondary outcome responses from 18 participants (T0 = 6, T1 = 12, T2 = 11). This meant that 87% of participants completed the proposed primary outcome measure post-intervention and 82.6% of participants completed the proposed primary outcome measure at six months (Figure 1). The completeness of each of the outcome measures was also high (Supplementary Table S1).

Summary statistics of each (sub)score across time are shown in Table 3. Results indicated improvement in all measures at either T1 or T2, or both except for disability (Table 4). Improvements in the fatigue measures were in line with published clinically meaningful changes.[33]

TABLE 3: SUMMARY OF RESPONSES WITH MEANS AND STANDARD DEVIATIONS AND RANGES

TABLE 4: MEAN DIFFERENCE BETWEEN TIME POINTS WITH 95% CONFIDENCE INTERVALS

Results from the health economic analysis are presented in Table 5. The key cost driver for this patient group was medication use, with very costly biologics driving the overall medication costs for some participants. Other substantial contributors to the overall cost from

the NHS/PSS perspective were hospital inpatient, outpatient and day cases. Care costs (both informal and privately paid) represented considerable cost burdens from the patient perspective. The mean delivery cost was estimated to be £98.40 per participant, rising to £128 when training costs were included.

TABLE 5: COSTS AND OUTCOMES PER PARTICIPANT USING ALL AVAILABLE DATA

DISCUSSION

During the FREE-IA study, RHPs delivered over 100 intervention sessions to patients struggling with the impact of fatigue. Results from the participant-reported outcomes suggest that this flexible, low-cost intervention has the potential to help patients self-manage this symptom. There is existing evidence for the effectiveness of higher intensity interventions delivered over several weeks to groups of patients.[14, 15] If the fatigue-related support needs of some patients could be met with a lower intensity intervention delivered over fewer sessions, it could increase choice and provision. The evidence that RHPs from different professional backgrounds undertook training and delivered the intervention further increases the possibility that this type of support could be practical to provide in a range of clinical settings. Although some sessions lasted for longer than the guideline of 20-30 minutes, most participants did not take up the maximum four sessions, with half attending three sessions and around 10% attending all four sessions. The intervention was estimated to be delivered at a relatively low cost per participant. Although the FREE-IA study sample is too small to evaluate whether duration and number of intervention sessions influenced outcomes, results suggest that 2-3 sessions might be enough for patients to derive clinically meaningful benefit.

An appropriate next step is to conduct a definitive RCT to test the clinical and cost-effectiveness of our intervention. This single-arm feasibility study explored several uncertainties and has provided insights to inform the design and delivery of such a study.

For peer review only - http://bmjopen.bmj.com/site/about/guidelines.xhtml

These include understanding variation in local processes and the resources available to support recruitment and intervention delivery, for example how to identify and invite potential participants and how to collect consent with minimal impact on the workload and time of RHPs. Collecting the proposed primary outcome by telephone and secondary outcomes via mail was a successful strategy overall. However, it was not always possible to contact participants by telephone or convenient for them to respond at that time. Returning paper outcomes in the mail might have been difficult, for example due to ‘shielding’ during the COVID-19 pandemic (namely, people who were advised not to leave their homes and to minimise all face-to-face contact). In a future study, we would seek ethics approval to incorporate options to contact participants by text and email and to collect outcomes online, as well as including the telephone and paper options. Improvements to the Resource Use Questionnaire (RUQ) were identified, allowing an optimised approach for a definitive RCT. The small number of audio-recorded sessions suggests that we need to find a different approach to evaluating competency and fidelity. Anecdotal feedback from RHPs suggests that gaining consent for audio-recording at the start of the intervention session took up too much time and audio-recording altered the interaction with participants, making it less like ‘real life’ clinical practice. We also need to reconsider the aim to deliver core session 2 within two weeks of core session 1, given that RHPs and/or patients were often unable to do this. Reasons for this were not systematically captured, but included difficulty booking and/or attending clinic appointments within the short timeframe. A key rationale for this timeframe was to review participants’ activity diaries, one of the intervention tools introduced in session 1 (Table 1). Options in the future include providing activity diaries to cover a longer period or having brief activity diary reviews by telephone between intervention sessions.

While our results suggest that a definitive RCT is feasible and our intervention has the potential to be helpful to patients, the large-scale changes in rheumatology care provision in response to the COVID-19 pandemic will impact the next steps.[34, 35] The move from face-to-face to telephone and video consultations is likely to result in long-term changes and has

For peer review only - http://bmjopen.bmj.com/site/about/guidelines.xhtml

implications for the testing and possible implementation of our intervention. However, the clear and careful design of FREE-IA mean that the training and intervention are well-positioned to be adapted for delivery in a range of modes and settings, including online. Although remote delivery of sessions was barely used in the current study, many patients and RHPs are becoming more familiar and comfortable with telephone and/or video interactions.[36, 37] In addition to influencing current practice, aspects of the intervention could inform professional pre-registration education programmes therefore helping another generation of NHS health professionals to support patients to self-manage their fatigue.

Study strengths include the low levels of attrition and the high levels of completed outcomes collected. Standardised outcome collection was ensured by the central team who were external to the hospitals delivering the intervention. As well as informing the design of a definitive RCT, our flexible, pragmatic approach to local variation meant that we gained insights into how the intervention could be delivered in clinical practice. This study benefitted from the input of two patient research partners, MU and BA, who contributed throughout the study, from identifying the research question through to interpreting the results. Feedback from the Patient Advisory Group of the Rheumatology Department at the Bristol Royal Infirmary also enhanced the study.

Study limitations include the lack of a control arm. To maximise information relating to the intervention itself, we did not include a concurrent control group and hence have not tested the feasibility/acceptability of randomisation. However, given that the intervention is not available in routine care, it is likely that patients willing to try the intervention, as in this study, are also likely to accept randomisation. This was a feasibility study and as such the data on health-related outcomes should not be over-interpreted: the improvements seen are within-patient comparisons only, hence could arise from regression to the mean or the small sample size. However, outcomes were in the direction to suggest the intervention could have

For peer review only - http://bmjopen.bmj.com/site/about/guidelines.xhtml

a beneficial impact on patients' fatigue, and confidence intervals support an interpretation of improvement.

CONCLUSIONS

We were able to design and deliver intervention training to RHPs, who were then able to deliver intervention sessions to participants, guided by the intervention manual. However, it was not always possible to deliver core session 2 within the desired two-week timeframe. We were able to collect outcomes at three time points and had low levels of attrition. Overall, our results suggest that a definitive RCT is feasible. While being cautious, outcomes were in a direction to suggest improvement in participants' fatigue impact after attending relatively low-cost intervention sessions.

For peer review only

CONTRIBUTORSHIP STATEMENT

Emma Dures: funding acquisition; study conceptualisation; methodology; study supervision; writing original draft

Susan Bridgewater: data collection; study management; data analysis; reviewed draft

Bryan Abbott: study conceptualisation; study delivery; reviewed draft

Jo Adams: study conceptualisation; reviewed draft

Alice Berry: data collection; data analysis; reviewed draft

Lance M McCracken: study conceptualisation; study delivery; reviewed draft

Siobhan Creanor: methodology; study delivery; formal data analysis; reviewed draft

Sarah Hewlett: study conceptualisation; methodology; study delivery; reviewed draft

Joe Lomax: formal data analysis; reviewed draft

Mwidimi Ndosi: methodology; reviewed draft

Joanna C Thorn: methodology; formal data analysis; reviewed draft

Marie Urban: study conceptualisation; study delivery; reviewed draft

Paul Ewings: methodology; formal data analysis; reviewed draft

COMPETING INTERESTS: none

FUNDING: this work was supported by a National Institute for Health Research (NIHR) grant under its research for Patient Benefit (RfPB) Programme [Grant Reference Number PB-PG-1216-20014]. This publication presents independent research funded by the NIHR. The views expressed are those of the authors and not necessarily those of the NHS, the NIHR or the Department of Health and Social Care.

DATA SHARING STATEMENT

Data will be available from the lead author on request.

For peer review only - http://bmjopen.bmj.com/site/about/guidelines.xhtml

References

1. Young, A., et al., *How does functional disability in early rheumatoid arthritis (RA) affect patients and their lives? Results of 5 years of follow-up in 732 patients from the Early RA Study (ERAS).* Rheumatology, 2000. **39**(6): p. 603-611.

2. Rheumatology, T.B.S.f. *Simple Tasks: Rheumatic conditions in the UK*. 2013; Available from: http://www.rheumatology.org.uk/includes/documents/cm_docs/2013/w/white_paper_report.pdf.

3. Homer, D., *Addressing psychological and social issues of rheumatoid arthritis within the consultation: a case report.* Musculoskeletal Care, 2005. **3**(1): p. 54-9.

4. Luqmani, R., et al., *British Society for Rheumatology and British Health Professionals in Rheumatology guideline for the management of rheumatoid arthritis (the first two years).* Rheumatology, 2006. **45**(9): p. 1167-1169.

5. Hewlett, S., et al., *Patients' perceptions of fatigue in rheumatoid arthritis: Overwhelming, uncontrollable, ignored.* Arthritis Care & Research, 2005. **53**(5): p. 697-702.

6. Overman, C.L., et al., *The prevalence of severe fatigue in rheumatic diseases: an international study.* Clin Rheumatol, 2016. **35**(2): p. 409-15.

7. Dures, E., et al., *Patients' Perspectives on the Psychological Impact of Inflammatory Arthritis and Meeting the Associated Support Needs: Open-Ended Responses in a Multi-Centre Survey.* Musculoskeletal Care, 2016.

8. Dures, E., et al., *Patient preferences for psychological support in inflammatory arthritis: a multicentre survey.* Ann Rheum Dis, 2016. **75**(1): p. 142-7.

9. Repping-Wuts, H., et al., *Fatigue in patients with rheumatoid arthritis: British and Dutch nurses' knowledge, attitudes and management.* J Adv Nurs, 2009. **65**(4): p. 901-11.

10. Geenen, R. and E. Dures, *A biopsychosocial network model of fatigue in rheumatoid arthritis: a systematic review.* Rheumatology, 2019. **58**(Supplement_5): p. v10-v21.

For peer review only - http://bmjopen.bmj.com/site/about/guidelines.xhtml

11. Almeida, C., et al., *Biologic interventions for fatigue in rheumatoid arthritis.* Cochrane Database Syst Rev, 2016(6): p. Cd008334.
12. Druce, K.L., et al., *Most patients who reach disease remission following anti-TNF therapy continue to report fatigue: results from the British Society for Rheumatology Biologics Register for Rheumatoid Arthritis.* Rheumatology (Oxford), 2016. **55**(10): p. 1786-90.
13. Cramp, F., et al., *Non-pharmacological interventions for fatigue in rheumatoid arthritis.* Cochrane Database Syst Rev, 2013(8): p. Cd008322.
14. Hewlett, S., et al., *Self-management of fatigue in rheumatoid arthritis: a randomised controlled trial of group cognitive-behavioural therapy.* Ann Rheum Dis, 2011. **70**(6): p. 1060-7.
15. Hewlett, S., et al., *Reducing arthritis fatigue impact: two-year randomised controlled trial of cognitive behavioural approaches by rheumatology teams (RAFT).* Ann Rheum Dis, 2019.
16. Ng, J.Y., et al., *Self-Determination Theory Applied to Health Contexts: A Meta-Analysis.* Perspect Psychol Sci, 2012. **7**(4): p. 325-40.
17. Bandura, A., *Social cognitive theory: an agentic perspective.* Annu Rev Psychol, 2001. **52**: p. 1-26.
18. White, C., in *Cognitive Behaviour Therapy for Chronic Medical Problems: A Guide to Assessment and Treatment in Practice.* 2001, Wiley: Chichester.
19. Craig, P., et al., *Developing and evaluating complex interventions: the new Medical Research Council guidance.* Int J Nurs Stud, 2013. **50**(5): p. 587-92.
20. Wood, S.J., et al., *Twelve tips to revitalise problem-based learning.* Med Teach, 2015. **37**(8): p. 723-729.
21. Nicklin, J., et al., *Measuring fatigue in rheumatoid arthritis: a cross-sectional study to evaluate the Bristol Rheumatoid Arthritis Fatigue Multi-Dimensional questionnaire, visual analog scales, and numerical rating scales.* Arthritis Care Res (Hoboken), 2010. **62**(11): p. 1559-68.

For peer review only
For peer review only - http://bmjopen.bmj.com/site/about/guidelines.xhtml

22. Gossec, L., et al., *Finalisation and validation of the rheumatoid arthritis impact of disease score, a patient-derived composite measure of impact of rheumatoid arthritis: a EULAR initiative.* Ann Rheum Dis, 2011. **70**(6): p. 935-42.

23. Pincus, T., et al., *Assessment of patient satisfaction in activities of daily living using a modified Stanford Health Assessment Questionnaire.* Arthritis Rheum, 1983. **26**(11): p. 1346-53.

24. Hewlett, S., et al., *Development and validation of a self-efficacy scale for use in British patients with rheumatoid arthritis (RASE).* Rheumatology (Oxford), 2001. **40**(11): p. 1221-30.

25. Dempster, M. and M. Donnelly, *Validity of the Perceived Health Competence Scale in a UK primary care setting.* Psychol Health Med, 2008. **13**(1): p. 123-7.

26. Williams, G.C., et al., *Autonomous regulation and long-term medication adherence in adult outpatients.* Health Psychol, 1998. **17**(3): p. 269-76.

27. Herdman, M., et al., *Development and preliminary testing of the new five-level version of EQ-5D (EQ-5D-5L).* Qual Life Res, 2011. **20**(10): p. 1727-36.

28. van Hout, B., et al., *Interim scoring for the EQ-5D-5L: mapping the EQ-5D-5L to EQ-5D-3L value sets.* Value Health, 2012. **15**(5): p. 708-15.

29. *National cost collection data*. Available from: https://www.england.nhs.uk/national-cost-collection/.

30. *Mileage allowances - Section 17* Available from: https://www.nhsemployers.org/pay-pensions-and-reward/agenda-for-change/nhs-terms-and-conditions-of-service-handbook/mileage-allowances/.

31. *Unit costs of health and social care 2019*. Available from: https://www.pssru.ac.uk/project-pages/unit-costs/unit-costs-2019/.

32. *BMA and Royal Pharmaceutical Society.* . 2019 ed. British National Formulary 76. London: BMJ Group and Pharmaceutical Press.

33. Dures, E.K., et al., *Reliability and sensitivity to change of the Bristol Rheumatoid Arthritis Fatigue scales.* Rheumatology (Oxford), 2013. **52**(10): p. 1832-9.

For peer review only - http://bmjopen.bmj.com/site/about/guidelines.xhtml

34. Bonfá, E., et al., *How COVID-19 is changing rheumatology clinical practice.* Nature Reviews Rheumatology, 2021. **17**(1): p. 11-15.

35. Sloan, M., et al., *The impact of the COVID-19 pandemic on the medical care and health-care behaviour of patients with lupus and other systemic autoimmune diseases: a mixed methods longitudinal study.* Rheumatology Advances in Practice, 2020. **5**(1).

36. Howren, A., et al., *Virtual rheumatology appointments during the COVID-19 pandemic: an international survey of perspectives of patients with rheumatic diseases.* Clinical Rheumatology, 2020. **39**(11): p. 3191-3193.

37. Kirby, T., *Rheumatologists rapidly adjust patient care during COVID-19 pandemic.* The Lancet Rheumatology, 2020. **2**(5): p. e258.

For peer review only

For peer review only - http://bmjopen.bmj.com/site/about/guidelines.xhtml

TABLE 1: OVERVIEW OF INTERVENTION STRUCTURE AND CONTENT

**Sessions 1-4**	**Key topics**	**Key handouts**
Engagement and validation	Identify fatigue drivers<br>Activity management	Fatigue overview<br>Activity diaries
Daily diary, goals, action planning	Boom and bust; avoidance and withdrawal<br>Drainers and energisers	Pacing<br>Goal setting<br>Activity diaries
Sleep and rest	Nature of sleep difficulties<br>Sleep myths and strategies	Sleep and relaxation<br>Activity diaries
Stress and relaxation	Symptoms of stress<br>Coping resources	Stress bucket<br>Activity diaries

For peer review only

TABLE 2: FREE-IA PARTICIPANT DEMOGRAPHICS

	**Study Participants (n = 46)**
**Sex (%)**	
Male	9 (19.6%)
Female	32 (69.6%)
Missing	5 (10.9%)
**Ethnicity (%)**	
White	39 (84.8%)
Black	1 (2.2%)
Prefer not to say	1 (2.2%)
Missing	5 (10.9%)
**Age in years (%)**	
< 40	5 (10.9%)
40 - 49	10 (21.7%)
50 - 59	15 (32.6%)
60 - 69	7 (15.2%)
70 - 79	3 (6.5%)
Missing	6 (13.0%)
**Site (%)**	
1 (South East England)	8 (17.4%)
2 (South East England)	7 (15.2%)
3 (South West England)	15 (32.6%)
4 (North West England)	10 (21.7%)
5 (South West England)	6 (13.0%)

For peer review only

TABLE 3: SUMMARY OF PARTICIPANT-REPORTED OUTCOME MEASURES WITH MEANS, STANDARD DEVIATIONS AND RANGES

Measure	Time Point 0	Time Point 1	Time Point 2
BRAF-NRS Fatigue Effect (0-10)	8.48 (1.19) (6.00,10.00) (n=46)	6.68 (1.54) (4.00, 9.00) (n=40)	6.03 (2.72) (0.00, 10.00) (n=39)
BRAF-NRS Coping (0-10)	6.68 (2.25) (1.00, 10.00) (n=41)	5.79 (2.53) (0.00, 10.00) (n=34)	5.03 (2.72) (0.00, 10.00) (n=34)
RAID Final Score (0-10)	6.40 (1.60) (1.87, 9.25) (n=41)	5.57 (2.00) (1.65, 8.79) (n=34)	5.54 (1.91) (1.30, 8.79) (n=36)
BRAF-MDQ Physical Severity (0-22)	17.92 (2.82) (11.00, 22.00) (n=41)	14.97 (4.16) (5.00, 22.00) (n=34)	14.56 (5.22) (4.00, 22.00) (n=34)
BRAF MDQ Living with Fatigue (0-21)	12.42 (4.95) (4.00, 21.00) (n=41)	9.09 (6.10) (0.00, 21.00) (n=34)	8.63 (5.88) (0.00, 21.00) (n=34)
BRAF-MDQ Cognitive (0-15)	9.39 (3.93) (1.00, 15.00) (n=41)	7.62 (3.82) (0.00, 15.00) (n=34)	7.09 (3.51) (1.00, 15.00) (n=34)
BRAF-MDQ Emotional (0-12)	7.71 (3.16) (1.00, 12.00) (n=41)	5.44 (3.51) (1.00, 12.00) (n=34)	5.47 (3.52) (0.00, 12.00) (n=34)
BRAF-MDQ Total (0-70)	47.43 (12.60) (21.00, 66.00) (n=41)	37.12 (15.39) (14.00, 68.00) (n=34)	35.75 (15.84) (9.00, 66.00) (n=34)
MHAQ Mean Score (0-4)	0.84 (0.58) (0.00, 2.38) (n=41)	0.72 (0.55) (0.00, 2.13) (n=33)	0.81 (0.61) (0.00, 2.00) (n=34)
HCCQ (1-7)*	3.95 (1.50) (1.17, 7.00) (n=39)	5.46 (1.36) (2.00, 7.00) (n=34)	4.85 (1.69) (1.33, 7.00) (n=36)
RASE (28-140)*	100.16 (12.20) (78.00, 128.00) (n=38)	105.67 (13.36) (72.00, 140.00) (n=33)	104.32 (16.21) (72.00, 135.00) (n=35)

** Higher scores indicates better outcome*

For peer review only

TABLE 4: MEAN DIFFERENCE BETWEEN TIME POINTS (BASELINE AND EACH FOLLOW UP) IN PARTICIPANT-REPORTED OUTCOME MEASURES WITH 95% CONFIDENCE INTERVALS

**Measure**	**T1-T0**	**T2-T0**
BRAF-NRS Fatigue Effect (0-10)	-1.78 (-2.27, -1.28) (n=40)	-2.41 (-3.29, -1.53) (n=39)
BRAF-NRS Coping (0-10)	-0.59 (-1.53, 0.34) (n=32)	-1.06 (-2.00, -0.12) (n=32)
RAID Final Score (0-10)	-0.64 (-1.27, -0.00) (n=32)	-0.61 (-1.32, 0.10) (n=33)
BRAF-MDQ Physical Severity (0-22)	-2.44 (-3.75, -1.12) (n=32)	-2.87 (-4.85, -0.89) (n=30)
BRAF-MDQ Living with Fatigue (0-21)	-2.75 (-4.52, -0.98) (n=32)	-2.72 (-4.55, -0.88) (n=30)
BRAF-MDQ Cognitive (0-15)	-1.84 (-3.19, -0.50) (n=32)	-1.63 (-3.22, -0.05) (n=30)
BRAF-MDQ Emotional (0-12)	-1.47 (-2.51, -0.42) (n=32)	-1.67 (-3.06, -0.27) (n=30)
BRAF-MDQ Total (0-70)	-8.50 (-13.03, -3.97) (n=32)	-8.88 (-15.00, -2.77) (n=30)
MHAQ Mean Score (0-4)	-0.07 (-0.23, 0.08) (n=31)	0.03 (-0.15, 0.21) (n=31)
HCCQ (1-7)	1.35 (0.65, 2.05) (n=31)	1.01 (0.35, 1.67) (n=32)
RASE (28-140)	3.32 (-0.62, 7.26) (n=31)	4.80 (1.00, 8.60) (n=32)

For peer review only - http://bmjopen.bmj.com/site/about/guidelines.xhtml

TABLE 5: COSTS AND OUTCOMES PER PARTICIPANT USING ALL AVAILABLE DATA

Table 5. Costs and outcomes per participant using all available data

	n	**Mean resource use**	**(SD)**	**Mean costs (£)**	**(SD)**	**95% CI**	
**Resource use**							
A&E visits	35	0.14	0.36	23.71	58.94		
Outpatient visits	30	1.43	1.76	210.70	258.05		
Day cases	30	0.40	1.33	300.80	999.20		
Inpatient stays	30	0.10	0.31	224.57	777.42		
GP appointments	34	1.94	2.37	66.00	80.69		
Nurse appointments	34	1.56	2.26	16.91	24.51		
GP home visits	30	0	0	0.00	0.00		
Nurse home visits	30	0.07	0.37	1.47	8.05		
Medications	30	2.57	1.41	2729.66	2796.45		
Nurse helpline	35	0.66	1.03	37.13	58.05		
Carer contacts	35	5.94	30.95	68.34	355.90		
**Total cost (NHS/PSS perspective)**				3690.08	3660.83	2323.10	5057.05
Informal care contacts	35	71.33	165.20	621.99	1440.58		
Private healthcare				82.33	180.38		
Private carers				128.03	365.83		
**Total cost (patient perspective)**				624.83	1072.68	224.28	1025.37
**Outcomes**							
	n	**Mean QALYs**	**(SD)**				
QALYs over the six month period	27	0.275	0.105			0.23	0.32

For peer review only

FREE-IA FLOW DIAGRAM OF PARTICIPANTS

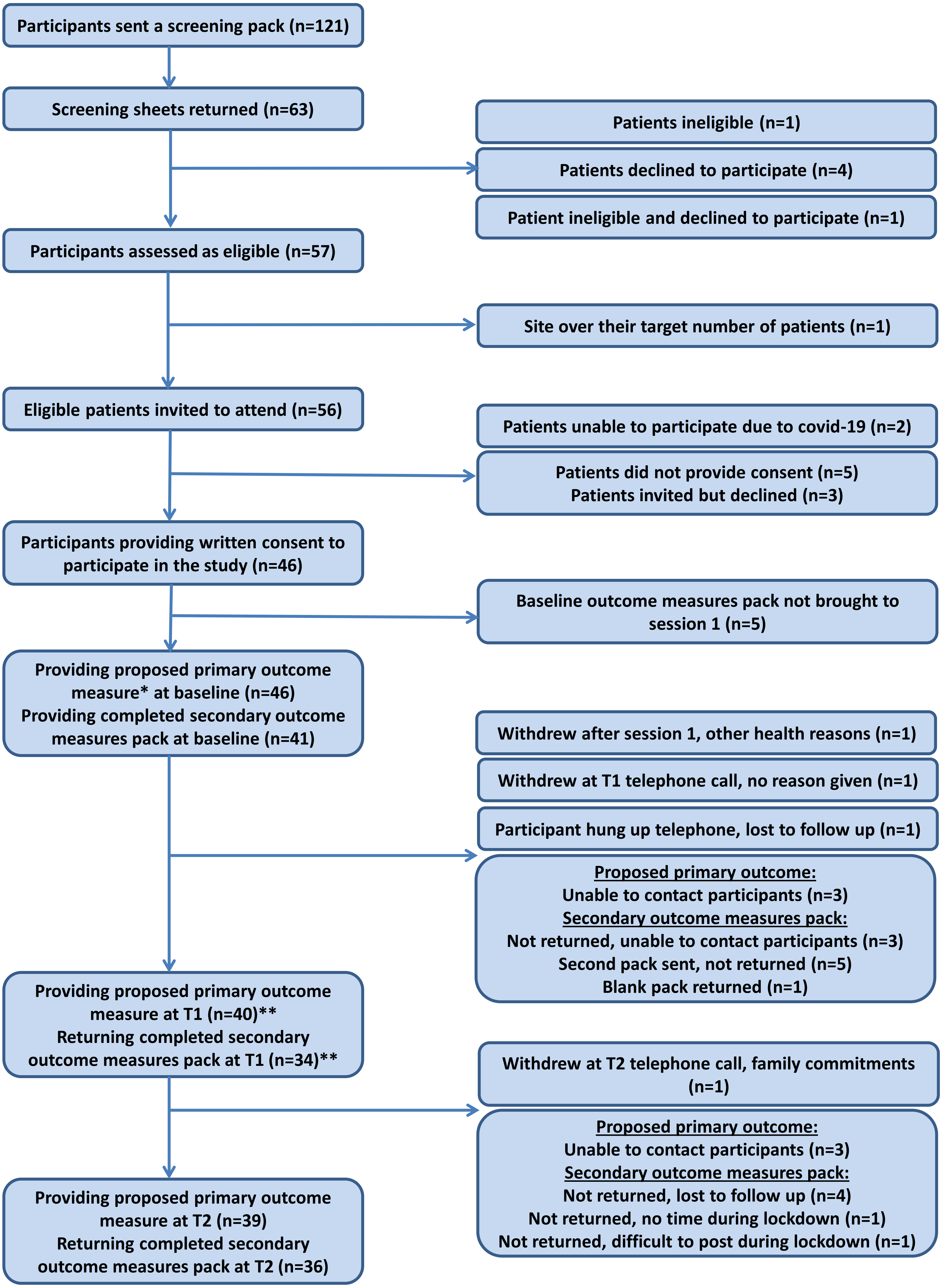

*The proposed primary outcome was collected by telephone

**Some participants did not return T1 outcomes but remained in the study and subsequently returned T2 outcomes

SUPPLEMENTARY TABLE S1 (PERCENTAGE OF COMPLETE/IMPUTED RESPONSES)

Measure	Time Point 0	Time Point 1	Time Point 2
BRAF-NRS Fatigue Effect (0-10)	100%	87.00%	84.78%
BRAF-NRS Coping (0-10)	89.13%	73.91%	73.91%
RAID Final Score (0-10)	89.13%	73.91%	73.91%
BRAF-MDQ Physical Severity (0-22)	89.13%	73.91%	73.91%
BRAF MDQ Living with Fatigue (0-21)	89.13%	73.91%	73.91%
BRAF-MDQ Cognitive (0-15)	89.13%	73.91%	73.91%
BRAF-MDQ Emotional (0-12)	89.13%	73.91%	73.91%
BRAF-MDQ Total (0-70)	89.13%	73.91%	73.91%
MHAQ Mean Score (0-4)	89.13%	71.74%	73.91%
HCCQ (1-7)	84.78%	73.91%	78.26%
RASE (28-140)	82.61%	71.74%	76.09%

For peer review only

BMJ Open

A brief intervention to reduce fatigue impact in patients with inflammatory arthritis: design and outcomes of a single-arm feasibility study

Journal:	*BMJ Open*
Manuscript ID	bmjopen-2021-054627.R1
Article Type:	Original research
Date Submitted by the Author:	16-Mar-2022
Complete List of Authors:	Dures, Emma; Bristol Royal Infirmary, Academic Rheumatology; University of the West of England<br>Bridgewater, Susan; University of the West of England<br>Abbott, Bryan ; Bristol Royal Infirmary<br>Adams, Jo; University of Southampton<br>Berry, Alice; University of the West of England<br>McCracken, Lance; Uppsala University<br>Creanor, Siobhan; University of Exeter<br>Hewlett, Sarah; University of the West of England<br>Lomax, Joe; Plymouth University<br>Ndosi, Mwidimi; Bristol Royal Infirmary, Academic Rheumatology Unit; University of the West of England, Nursing and Midwifery<br>Thorn, Joanna; University of Bristol<br>Urban, Marie; Bristol Royal Infirmary<br>Ewings, Paul; Taunton and Somerset NHS Foundation Trust
**Primary Subject Heading**:	Rheumatology
Secondary Subject Heading:	Patient-centred medicine
Keywords:	MEDICAL EDUCATION & TRAINING, REHABILITATION MEDICINE, RHEUMATOLOGY

SCHOLARONE™
Manuscripts

BMJ

I, the Submitting Author has the right to grant and does grant on behalf of all authors of the Work (as defined in the below author licence), an exclusive licence and/or a non-exclusive licence for contributions from authors who are: i) UK Crown employees; ii) where BMJ has agreed a CC-BY licence shall apply, and/or iii) in accordance with the terms applicable for US Federal Government officers or employees acting as part of their official duties; on a worldwide, perpetual, irrevocable, royalty-free basis to BMJ Publishing Group Ltd ("BMJ") its licensees and where the relevant Journal is co-owned by BMJ to the co-owners of the Journal, to publish the Work in this journal and any other BMJ products and to exploit all rights, as set out in our licence.

The Submitting Author accepts and understands that any supply made under these terms is made by BMJ to the Submitting Author unless you are acting as an employee on behalf of your employer or a postgraduate student of an affiliated institution which is paying any applicable article publishing charge ("APC") for Open Access articles. Where the Submitting Author wishes to make the Work available on an Open Access basis (and intends to pay the relevant APC), the terms of reuse of such Open Access shall be governed by a Creative Commons licence – details of these licences and which Creative Commons licence will apply to this Work are set out in our licence referred to above.

Other than as permitted in any relevant BMJ Author's Self Archiving Policies, I confirm this Work has not been accepted for publication elsewhere, is not being considered for publication elsewhere and does not duplicate material already published. I confirm all authors consent to publication of this Work and authorise the granting of this licence.

For peer review only

A brief intervention to reduce fatigue impact in patients with inflammatory arthritis: design and outcomes of a single-arm feasibility study

Emma Dures[1,2], Susan Bridgewater[1,2], Bryan Abbott[2], Jo Adams[3], Alice Berry[1,2], Lance M McCracken[4], Siobhan Creanor[5], Sarah Hewlett[2], Joe Lomax[6], Mwidimi Ndosi[1,2], Joanna C Thorn[7], Marie Urban[2], Paul Ewings[8]

Author affiliations

[1]Academic Rheumatology, University Hospitals Bristol and Weston NHS Foundation Trust, Bristol, UK

[2]Faculty of Health and Applied Sciences, University of the West of England, Bristol, UK

[3]Health Sciences, University of Southampton, Southampton, UK

[4]Department of Psychology, Uppsala University, Uppsala, Sweden

[5]Exeter Clinical Trials Unit, College of Medicine & Health, University of Exeter, UK

[6]Medical Statistics Group, Faculty of Health, University of Plymouth, Plymouth, UK

[7]Bristol Medical School, University of Bristol, Bristol, UK

[8]Department of Research, Somerset NHS Foundation Trust, Taunton, UK

Corresponding author

Emma Dures

Academic Rheumatology

The Bristol Royal Infirmary

Bristol BS2 8HW

UK

emma2.dures@uwe.ac.uk

For peer review only

ABSTRACT

Objectives

Patients with inflammatory arthritis report that fatigue is challenging to manage. We developed a manualised, one-to-one, cognitive-behavioural intervention, delivered by rheumatology health professionals (RHPs). The FREE-IA (Fatigue - Reducing its Effects through individualised support Episodes in Inflammatory Arthritis) study tested the feasibility of RHP training, intervention delivery, and outcome collection, ahead of a potential trial of clinical and cost-effectiveness.

Methods

In this single-arm feasibility study, eligible patients were ≥18 years, had a clinician-confirmed diagnosis of an inflammatory arthritis and scored ≥6/10 on the BRAF NRS Fatigue Effect. Following training, RHPs delivered 2–4 sessions to participants. Baseline data were collected before the first session (T0), and outcomes at six weeks (T1) and six months (T2). The proposed primary outcome was fatigue impact (BRAF NRS Fatigue Effect). Secondary outcomes included fatigue severity and coping, disease impact and disability, and measures of therapeutic mechanism (self-efficacy and confidence to manage health).

Results

Eight RHPs at five hospitals delivered 113 sessions to 46 participants. Of a potential 138 primary and secondary outcome responses at T0, T1 and T2, there were 13 (9.4%) and 27 (19.6%) missing primary and secondary outcome responses, respectively. Results indicated improvements in all measures except disability, at either T1 or T2, or both.

Conclusions

This study showed it was feasible to deliver the intervention, including training RHPs, and recruit and follow-up participants with high retention. While there was no control group, observed within-group improvements suggest potential promise of the intervention and support for a definitive trial to test effectiveness.

For peer review only - http://bmjopen.bmj.com/site/about/guidelines.xhtml

Strengths and limitations of this study

- This feasibility study has established that rheumatology health professionals can train and deliver a brief, low-cost intervention for fatigue in inflammatory arthritis.
- The low levels of attrition and high levels of data completeness suggest the outcomes collected are appropriate for a definitive trial.
- Within-group improvements were observed, although this could have arisen from regression to the mean or the small sample size.
- The lack of a control arm means that the feasibility/acceptability of randomisation has not been tested.

For peer review only

INTRODUCTION

Inflammatory arthritis (IA) is a group of multi-systemic, auto-immune conditions characterised by pain, joint swelling and stiffness, and fatigue. The most common of these conditions is rheumatoid arthritis (RA).[1] It is estimated that over 750,000 adults in the United Kingdom (UK) have an IA.[2, 3] Challenges for patients with IA include unpredictable fluctuations in symptoms, functional disability, and managing complex medication regimens.[4] Treatment options include pharmacological, non-pharmacological, and surgical interventions to control symptoms, prevent joint damage and improve mobility and function.[5] In the UK to date, treatment for IA is typically provided in secondary care by multi-disciplinary rheumatology health professionals (RHPs), including physicians, nurse specialists, occupational therapists, and physiotherapists.

Although the clinical manifestations vary, fatigue is a prevalent and often disabling symptom across types of IA [6-8] It is experienced by patients as a fluctuating, unpredictable symptom that impacts on all aspects of daily life. [9] An international study of >6,000 IA patients found that one out of every two was severely fatigued, defined as scoring ≤35 on the SF-36 Vitality Scale.[10] Despite the high prevalence and impact of the symptom, patients perceive that often their fatigue is not addressed in rheumatology consultations.[11] UK research with >1,200 IA patients found that 82% wanted support to manage the impact of pain and fatigue.[12] RHPs have reported that they recognise that fatigue is an issue for patients but there is a lack of evidence-based resources that they can use in clinical practice.[13]

Fatigue is a complex, multi-faceted phenomenon, the mechanisms of which are not fully understood. Challenges include the difficulty of measuring fatigue, and the high number of previous studies that have used cross-sectional designs, making it hard to understand directionality and attribute causality.[6] However, from the evidence available, fatigue in IA is associated with inflammation, pain, disability, sleep, depression and health beliefs, implying complex, multi-causal pathways.[14] A systematic review found that biologic treatments in

For peer review only

patients with active RA can lead to a small to moderate improvement in their fatigue, suggesting that optimal disease activity management should be part of fatigue management.[15] However, biologic treatments are not prescribed for IA-related fatigue and there is evidence that patients can experience fatigue during remission.[16] A systematic review for non-pharmacological interventions concluded that physical activity and psychosocial interventions, including cognitive-behavioural therapy (CBT), provide benefit in relation to self-reported fatigue in adults with RA.[17] This evidence has underpinned several CBT-based self-management interventions for fatigue.[18, 19] Although clinically effective they are highly structured, stand-alone interventions comprising at least six patient contact sessions. Consequently, they are time-consuming for patients to attend and for RHPs to deliver.

In response, we developed the FREE-IA (Fatigue - Reducing its Effects through individualised support Episodes in Inflammatory Arthritis) study. As part of the study, we designed a brief, one-to-one intervention that aims to reduce fatigue impact by supporting patients to identify the thoughts, feelings and behaviours perpetuating their fatigue Supplementary Summary 1). Patients can then use this understanding as the basis for making adaptive behaviour changes and enhancing their coping skills. The intervention is based on self-determination theory, which addresses motivation and competence to behave in effective and healthy ways; self-efficacy, a belief in one's ability to successfully engage in a course of action; and guided discovery (the 'Ask don't tell' approach rather than didactic information and advice-giving).[20-22] The intervention was designed by a multi-disciplinary team from nursing (SH), occupational therapy (JA) and psychology (LM, ED) and written as a manual, designed to be used after training in cognitive-behavioural approaches, daily dairies and goal setting. It comprises 2-4 sessions, each designed to last 20-30 minutes (Table 1). The first two sessions are core and designed to take place face-to-face and within two weeks. Up to two additional optional sessions can take place face-to-face or remotely, for example by telephone or video, within the subsequent four weeks.

For peer review only - http://bmjopen.bmj.com/site/about/guidelines.xhtml

TABLE 1: OVERVIEW OF INTERVENTION STRUCTURE AND CONTENT

Our study design was informed by the Medical Research Council's framework for developing and evaluating complex interventions.[23] Before investing in a definitive randomised controlled trial (RCT) to test an intervention's clinical and cost effectiveness (evaluation stage), the research team should have a reasonable expectation that the intervention could have a worthwhile effect, based on existing evidence and theory (development stage). They should also examine whether the evaluation procedures are likely to be deliverable and acceptable (feasibility stage). Researchers are advised to use a mix of quantitative and qualitative methods to resolve the main uncertainties that might impede study delivery. To achieve this, we designed the feasibility study FREE-IA (Fatigue - Reducing its Effects through individualised support Episodes in Inflammatory Arthritis).

Our aims were to:

- design and deliver intervention training to RHPs;
- recruit patients to the intervention;
- determine the completeness of outcome measurement data collection from patients who participated in intervention sessions;
- and identify the optimum approach for a cost-effectiveness evaluation to be conducted alongside a definitive RCT.

We also examined the acceptability of the intervention from the perspectives of patients who participated and RHPs who undertook training and delivery, via telephone interviews. These data are reported separately in a qualitative process evaluation.

ETHICS APPROVAL STATEMENT

For peer review only

Ethics approvals for the study were granted by the South West - Frenchay Research Ethics (REC ref. 15/SW/0207). All participants provided written informed consent prior to taking part in the study.

MATERIALS AND METHODS

We used a single-arm feasibility study design comprising three phases:

- Phase 1: delivery of intervention training to RHPs
- Phase 2: patient recruitment and intervention delivery
- Phase 3: data collection and analysis

Phase I: we developed and delivered intervention training face-to-face. We included overviews of the IA fatigue evidence-base, underpinning psychological theories, and materials from the manual (cognitive components); skills demonstrations from the training team (modelling/illustrational component); skills practice using rheumatology-specific vignettes, with observation and feedback from the training team (experiential/behavioural component); and a problem-based learning approach, with RHPs using examples from their clinical practice.[24] Training was designed and delivered by ED, SH, LM and patient research partners MU and BA.

Phase II: individual secondary care sites made local decisions about their optimum strategy to invite patients to participate in the study. Eligibility criteria were rheumatology patients at a participating site; age 18 years and over with a clinician-confirmed diagnosis of IA; with a score ≥6/10 on the BRAF NRS Fatigue Effect[25] and with fatigue that they considered recurrent, frequent, and/or persistent; and who were not accessing support for their fatigue at the time of invitation. Patients who were unable to complete questionnaires in English unaided and/or patients lacking capacity to give informed consent were not eligible. Patients interested in participating completed and mailed their screening sheet to the study

For peer review only - http://bmjopen.bmj.com/site/about/guidelines.xhtml

coordinator SB, who assessed their eligibility for the study. Following confirmation of eligibility, SB mailed a baseline data pack to patients who were interested in taking part. The pack comprised a consent form, and a questionnaire to collect demographic and clinical data and the proposed outcome measures to be used in the definitive RCT (see phase III). SB asked patients to complete the baseline data pack, including the consent form, and to bring it to their first face-to-face intervention session.

After training, RHPs delivered intervention sessions to recruited patients. To inform patterns of uptake, amendments to the intervention and the cost of delivery, we asked RHPs to record the number and duration of intervention sessions delivered to each participant and the mode of delivery, for example face-to-face, by telephone or by video. Once they had experience of delivery, we asked RHPs to audio-record the intervention sessions, if the participant consented, to assess how the intervention was delivered. We designed a pro-forma to guide assessment of competence and fidelity to the intervention. It comprised two parts: (i) inclusion of intervention content/topics and (ii) use of facilitative approaches by the RHP. In each section, research fellow AB scored the extent to which planned content was present (0 = not present, + = attempted/present, ++ = present/a key focus) and made notes to include examples and reflections. This information was for process evaluation purposes (to be reported separately) and not as feedback for the RHPs delivering the intervention.

For peer review only

Phase III: after baseline (T0), we collected quantitative outcomes data from participants at two time-points: six weeks post-intervention (T1) and six months post-intervention (T2). We defined post-intervention as six weeks after core session 1 because it covered the maximum intended period of exposure to the intervention. Our likely primary outcome in a future RCT is fatigue impact, measured using the BRAF-NRS Fatigue Effect.[25] We also collected validated secondary outcomes:

- BRAF-NRS Fatigue Severity[25]
- BRAF-NRS Fatigue Coping[25]

For peer review only - http://bmjopen.bmj.com/site/about/guidelines.xhtml

- Rheumatoid Arthritis Impact of Disease (RAID)[26] (pain, functional disability, fatigue, sleep, coping, physical and emotional well-being)
- BRAF Multi-dimensional Questionnaire (BRAF-MDQ)[25]
- Modified Health Assessment Questionnaire (MHAQ)[27] (functional disability)

Measures of therapeutic mechanism:

- The Rheumatoid Arthritis Self-Efficacy Scale (RASE)[28] (beliefs reflecting confidence in one's capacity to function despite symptoms)
- The Perceived Health Competence Scale (PHCS)[29] (feelings of capability to manage health outcomes)
- The Health Care Climate Questionnaire (HCCQ)[30] (perceptions of the extent to which a health professional is autonomy supportive)

SB collected the proposed primary outcome by telephone and the secondary outcomes via an outcome measures pack that was mailed to participants at T1 and T2. Participants were asked to complete the questionnaires and mail them back.

The FREE-IA Project Management Group approved analysis plans for the statistical outcomes and health economics. Methodologists PE, JL and SC conducted analysis of the statistical outcomes. For each self-reported questionnaire, the total scale and subscale scores were calculated in line with published guidance, including the use of imputation for unanswered questions (Supplementary Table S1). Outcome scores are reported as means and standard deviations, plus ranges, at each of the three time points. In addition, the mean change from T0 to T2 for each (sub)scale, with 95% confidence intervals, is presented.

Health economic outcomes were analysed by health economist JT. Health-related quality of life (EQ-5D-5L)[31] was collected at T0, T1 and T2, and valued using the van Hout crosswalk method based on UK population preferences.[32] Mean quality-adjusted life years

For peer review only - http://bmjopen.bmj.com/site/about/guidelines.xhtml

(QALYs) were calculated over the six months of follow-up. A bespoke resource-use questionnaire was developed in consultation with patient partners, covering (1) NHS & personal social services (PSS) and (2) patient perspectives. An estimate of the cost of delivering the intervention itself was derived from study records. Standard sources were used to assign unit costs (2019) to each of the resources measured [33-36] and mean usage (e.g., appointments), mean costs and standard deviations were calculated over the six months of follow-up using all available cases. A non-comparative cost–consequences matrix was constructed.

PATIENT AND PUBLIC INVOLVEMENT

The research study, including the question of whether it would be feasible to train RHPs and deliver the intervention, was developed with patient research partners BA and MU, who have experience of living with IA and fatigue. They were co-applicants in the funding application and are co-authors on this manuscript. The proposal was also discussed with the Patient Advisory Group in the Rheumatology Department of the Bristol Royal Infirmary. BA and MU reviewed all patient-facing literature, shaped the bespoke health economics questionnaire, supported delivery of the intervention training, provided additional materials for RHPs delivering the intervention, advised on recruitment and helped to interpret the study findings. After study completion, they reviewed the written summaries that were sent to study participants, including patients and RHPs who had taken part.

ANALYSIS AND RESULTS

Delivery of intervention training to RHPs

We delivered face-to-face training three times, with different RHPs each time. In total, 12 RHPs (eight nurses, two occupational therapists, one associate rheumatology practitioner, and one clinical research practitioner) from six hospitals attended. The first training took place over two days at the hospital where the central study team are based, with seven RHPs from four sites and lasted for approx. 13 hours. Subsequently, one site withdrew from

For peer review only - http://bmjopen.bmj.com/site/about/guidelines.xhtml

the study after their two RHPs had attended training but before recruiting patients, due to logistical challenges of intervention delivery at their hospital. Subsequently, two new sites joined the study, with training delivered over one and a half days (approx.10 hours) at the same central study team hospital to four RHPs. The third training lasted for one day (approx. five hours) and was delivered by ED at the hospital of an individual RHP from one of the new sites who had been unable to attend the group session with colleagues.

Patient recruitment and intervention delivery

A total of 46 patients were recruited to the FREE-IA study (Figure 1, Table 2). The overall recruitment rate was 0.22 participants per hospital per month, however, most sites did not recruit continuously over the duration of the recruitment period. The conversion rate, based on the number of participants recruited divided by the number screened, was 52.1% (63/121). Six of the 63 patients (9.5%) who expressed interest in participating were ineligible and/or declined to participate. Of the remaining 57 patients, five did not provide consent (8.8%) and three declined an invitation to take part (5.3%). One site did not invite an eligible patient because they had reached their target recruitment and one site stopped recruitment early due to COVID-19, with the local team unable to invite two interested and eligible patients to participate in the study. This left 46 patients who provided written consent and who provided a proposed primary outcome at baseline.

FIGURE 1: FLOW DIAGRAM

Eight RHPs delivered 113 intervention sessions across five sites and duration ranged from 10-120 minutes (mean 44 minutes). One RHP took consent but did not deliver the intervention. At two sites, all intervention sessions were delivered by one RHP. At the three other sites, the number of intervention sessions delivered by each RHP varied. Of the total 46 participants, 39 (84.8%) completed the two core sessions. Seven (15.2%) attended one session, 16 (34.8%) attended two sessions, 18 (39.1%) attended three sessions, and five

For peer review only - http://bmjopen.bmj.com/site/about/guidelines.xhtml

(10.9%) attended the maximum four possible sessions. Mode of delivery was face-to-face, except for four optional sessions, which were delivered by telephone. Session 2 of the intervention was delivered within the desired two-week timeframe for 37% of the participants who attended at least the two core sessions, with a mean of 21 days between sessions. No adverse events were reported.

Twenty-five intervention sessions were audio-recorded across three sites; two sites did not record any sessions. AB evaluated all the audio-recordings and SB and ED analysed a sub-set independently. There was a high level of agreement between the team members in relation to the audio-recordings that were analysed in triplicate. The main insights were that:

- Most RHPs followed the manual in a highly structured, linear way, but some adopted a more flexible approach guided by patients' fatigue-related support needs.
- RHPs used the materials to prompt discussion using a non-didactic approach, initially to explore fatigue drivers and daily diaries, and later to explore goal setting, sleep, and stress.
- When it was difficult for patients to identify unhelpful behaviour patterns, some RHPs were more directive.
- Longer appointments allowed for linking thoughts and feelings with behaviours, developing goals, and exploring behaviour patterns.
- RHPs who had more time and/or experience and/or knew the patient from previous clinical appointments tended to explore negativity towards change with more confidence.

TABLE 2: FREE-IA PARTICIPANT DEMOGRAPHICS

<u>Data completeness and summary of patient-reported outcome measures</u>

For peer review only - http://bmjopen.bmj.com/site/about/guidelines.xhtml

There were 13 (9.4%) missing proposed primary outcome responses from 11 participants (T0 = 0, T1 = 6, T2 = 8) and 27 (19.6%) missing secondary outcome responses from 18 participants (T0 = 6, T1 = 12, T2 = 11). This meant that 87% of participants completed the proposed primary outcome measure post-intervention and 82.6% of participants completed the proposed primary outcome measure at six months (Figure 1). The completeness of each of the outcome measures was also high (Supplementary Table S1).

Summary statistics of each (sub)score across time are shown in Table 3. Results indicated improvement in all measures at either T1 or T2, or both except for disability (Table 4). Improvements in the fatigue measures were in line with published clinically meaningful changes.[37]

TABLE 3: SUMMARY OF PARTICIPANT-REPORTED OUTCOMES MEASURES AT ALL TIME POINTS AND MEAN DIFFERENCES WITH CORRESPONDING 95% CONFIDENCE INTERVALS

Results from the health economic analysis are presented in Table 4. The key cost driver for this patient group was medication use, with very costly biologics driving the overall medication costs for some participants. Other substantial contributors to the overall cost from the NHS/PSS perspective were hospital inpatient, outpatient and day cases. Care costs (both informal and privately paid) represented considerable cost burdens from the patient perspective. The mean delivery cost was estimated to be £98.40 per participant, rising to £128 when training costs were included.

TABLE 4: ECONOMIC EVALUATION MEASURES: RESOURCES USE, COSTS AND OUTCOMES OVER SIX MONTHS OF FOLLOW-UP

DISCUSSION

For peer review only - http://bmjopen.bmj.com/site/about/guidelines.xhtml

During the FREE-IA study, RHPs delivered over 100 intervention sessions to patients struggling with the impact of fatigue. Results from the participant-reported outcomes suggest that this flexible, low-cost intervention has the potential to help patients self-manage this symptom. There is existing evidence for the effectiveness of higher intensity interventions delivered over several weeks to groups of patients.[18, 19] If the fatigue-related support needs of some patients could be met with a lower intensity intervention delivered over fewer sessions, it could increase choice and provision. The evidence that RHPs from different professional backgrounds undertook training and delivered the intervention further increases the possibility that this type of support could be practical to provide in a range of clinical settings. Although some sessions lasted for longer than the guideline of 20-30 minutes, most participants did not take up the maximum four sessions, with half attending three sessions and around 10% attending all four sessions. The intervention was estimated to be delivered at a relatively low cost per participant. Although the FREE-IA study sample is too small to evaluate whether duration and number of intervention sessions influenced outcomes, results suggest that 2-3 sessions might be enough for patients to derive clinically meaningful benefit.

An appropriate next step is to design and conduct a definitive RCT to test the clinical and cost-effectiveness of our intervention. This single-arm feasibility study explored several uncertainties and has provided insights to inform the design and delivery of such a study. These include understanding variation in local processes and the resources available to support recruitment and intervention delivery, for example how to identify and invite potential participants and how to collect consent with minimal impact on the workload and time of RHPs. Collecting the proposed primary outcome by telephone and secondary outcomes via mail was a successful strategy overall. However, it was not always possible to contact participants by telephone or convenient for them to respond at that time. Returning paper outcomes in the mail might have been difficult, for example due to ‘shielding’ during the COVID-19 pandemic (namely, people who were advised not to leave their homes and to

For peer review only - http://bmjopen.bmj.com/site/about/guidelines.xhtml

minimise all face-to-face contact). In a future study, we would seek ethics approval to incorporate options to contact participants by text and email and to collect outcomes online, as well as including the telephone and paper options. Improvements to the Resource Use Questionnaire (RUQ) were identified, allowing an optimised approach for a definitive RCT. The small number of audio-recorded sessions suggests that we need to find a different approach to evaluating competency and fidelity. Anecdotal feedback from RHPs suggests that gaining consent for audio-recording at the start of the intervention session took up too much time and audio-recording altered the interaction with participants, making it less like 'real life' clinical practice. We also need to reconsider the aim to deliver core session 2 within two weeks of core session 1, given that RHPs and/or patients were often unable to do this. Reasons for this were not systematically captured but included difficulty booking and/or attending clinic appointments within the short timeframe. A key rationale for this timeframe was to review participants' activity diaries, one of the intervention tools introduced in session 1 (Table 1). Options in the future include providing activity diaries to cover a longer period or having brief activity diary reviews by telephone between intervention sessions.

While our results suggest that a definitive RCT is feasible and our intervention has the potential to be helpful to patients, the large-scale changes in rheumatology care provision in response to the COVID-19 pandemic will impact the next steps.[38, 39] The move from face-to-face to telephone and video consultations is likely to result in long-term changes and has implications for the testing and possible implementation of our intervention. However, the clear and careful design of FREE-IA mean that the training and intervention are well-positioned to be adapted for delivery in a range of modes and settings, including online. Although remote delivery of sessions was barely used in the current study, many patients and RHPs are becoming more familiar and comfortable with telephone and/or video interactions.[40, 41] In addition to influencing current practice, aspects of the intervention could inform professional pre-registration education programmes therefore helping another generation of NHS health professionals to support patients to self-manage their fatigue.

For peer review only - http://bmjopen.bmj.com/site/about/guidelines.xhtml

Study strengths include the low levels of attrition and the high levels of completed outcomes collected. Standardised outcome collection was ensured by the central team who were external to the hospitals delivering the intervention. As well as informing the design of a definitive RCT, our flexible, pragmatic approach to local variation meant that we gained insights into how the intervention could be delivered in clinical practice. This study benefitted from the input of two patient research partners, MU and BA, who contributed throughout the study, from identifying the research question through to interpreting the results. Feedback from the Patient Advisory Group of the Rheumatology Department at the Bristol Royal Infirmary also enhanced the study.

Study limitations include the lack of a control arm. To maximise information relating to the intervention itself, and given limited resources, we did not include a concurrent control group and hence have not tested the feasibility/acceptability of randomisation. However, given that the intervention is not available in routine care, it is likely that patients willing to try the intervention, as in this study, are also likely to accept randomisation. This was a feasibility study and as such the data on health-related outcomes should not be over-interpreted: the improvements seen are within-patient comparisons only, hence could arise from regression to the mean or the small sample size. However, outcomes were in the direction to suggest the intervention could have a beneficial impact on patients' fatigue, and confidence intervals support an interpretation of improvement. Our proposed primary outcome is the BRAF-NRS Fatigue Effect which was developed with patients who have RA, although it has subsequently been validated in patients with psoriatic arthritis.[42] There might also be other important outcomes, such as work productivity, that we could include in a future trial.

CONCLUSIONS

We were able to design and deliver intervention training to RHPs, who were then able to deliver intervention sessions to participants, guided by the intervention manual. However, it

For peer review only - http://bmjopen.bmj.com/site/about/guidelines.xhtml

was not always possible to deliver core session 2 within the desired two-week timeframe. We were able to collect outcomes at three time points and had low levels of attrition. Overall, our results suggest that a definitive RCT is feasible. While being cautious, outcomes were in a direction to suggest improvement in participants' fatigue impact after attending relatively low-cost intervention sessions.

For peer review only

For peer review only - http://bmjopen.bmj.com/site/about/guidelines.xhtml

CONTRIBUTORSHIP STATEMENT

Emma Dures: funding acquisition; study conceptualisation; methodology; study supervision; writing original draft

Susan Bridgewater: data collection; study management; data analysis; reviewed draft

Bryan Abbott: study conceptualisation; study delivery; reviewed draft

Jo Adams: study conceptualisation; reviewed draft

Alice Berry: data collection; data analysis; reviewed draft

Lance M McCracken: study conceptualisation; study delivery; reviewed draft

Siobhan Creanor: methodology; study delivery; formal data analysis; reviewed draft

Sarah Hewlett: study conceptualisation; methodology; study delivery; reviewed draft

Joe Lomax: formal data analysis; reviewed draft

Mwidimi Ndosi: methodology; reviewed draft

Joanna C Thorn: methodology; formal data analysis; reviewed draft

Marie Urban: study conceptualisation; study delivery; reviewed draft

Paul Ewings: methodology; formal data analysis; reviewed draft

COMPETING INTERESTS: none

FUNDING: this work was supported by a National Institute for Health Research (NIHR) grant under its research for Patient Benefit (RfPB) Programme [Grant Reference Number PB-PG-1216-20014]. This publication presents independent research funded by the NIHR. The views expressed are those of the authors and not necessarily those of the NHS, the NIHR or the Department of Health and Social Care.

DATA SHARING STATEMENT

Data will be available from the lead author on request.

For peer review only - http://bmjopen.bmj.com/site/about/guidelines.xhtml

References

1. Young, A., et al., *How does functional disability in early rheumatoid arthritis (RA) affect patients and their lives? Results of 5 years of follow-up in 732 patients from the Early RA Study (ERAS).* Rheumatology, 2000. **39**(6): p. 603-611.
2. *Simple Tasks: Rheumatic conditions in the UK*. 2013; Available from: http://www.rheumatology.org.uk/includes/documents/cm_docs/2013/w/white_paper_report.pdf.
3. *Versus Arthritis.* State of musculoskeletal health 2021; Available from: https://www.versusarthritis.org/about-arthritis/data-and-statistics/the-state-of-musculoskeletal-health/
4. Homer, D., *Addressing psychological and social issues of rheumatoid arthritis within the consultation: a case report.* Musculoskeletal Care, 2005. **3**(1): p. 54-9.
5. Luqmani, R., et al., *British Society for Rheumatology and British Health Professionals in Rheumatology guideline for the management of rheumatoid arthritis (the first two years).* Rheumatology, 2006. **45**(9): p. 1167-1169.
6. Davies, K., E. Dures, and W.F. Ng, *Fatigue in inflammatory rheumatic diseases: current knowledge and areas for future research.* Nat Rev Rheumatol, 2021. **17**(11): p. 651-664.
7. Dures, E., et al., *Fatigue in inflammatory arthritis.* Best Pract Res Clin Rheumatol, 2020. **34**(2): p. 101526.
8. Hewlett, S., et al., *Patients' perceptions of fatigue in rheumatoid arthritis: Overwhelming, uncontrollable, ignored.* Arthritis Care & Research, 2005. **53**(5): p. 697-702.
9. Primdahl, J., et al., *The Experience of people with rheumatoid arthritis living with fatigue: a qualitative metasynthesis.* BMJ Open, 2019. **9**(3): p. e024338.
10. Overman, C.L., et al., *The prevalence of severe fatigue in rheumatic diseases: an international study.* Clin Rheumatol, 2016. **35**(2): p. 409-15.
11. Dures, E., et al., *Patients' Perspectives on the Psychological Impact of Inflammatory Arthritis and Meeting the Associated Support Needs: Open-Ended Responses in a Multi-Centre Survey.* Musculoskeletal Care, 2016.
12. Dures, E., et al., *Patient preferences for psychological support in inflammatory arthritis: a multicentre survey.* Ann Rheum Dis, 2016. **75**(1): p. 142-7.
13. Repping-Wuts, H., et al., *Fatigue in patients with rheumatoid arthritis: British and Dutch nurses' knowledge, attitudes and management.* J Adv Nurs, 2009. **65**(4): p. 901-11.
14. Geenen, R. and E. Dures, *A biopsychosocial network model of fatigue in rheumatoid arthritis: a systematic review.* Rheumatology, 2019. **58**(Supplement_5): p. v10-v21.
15. Almeida, C., et al., *Biologic interventions for fatigue in rheumatoid arthritis.* Cochrane Database Syst Rev, 2016(6): p. Cd008334.
16. Druce, K.L., et al., *Most patients who reach disease remission following anti-TNF therapy continue to report fatigue: results from the British Society for Rheumatology Biologics Register for Rheumatoid Arthritis.* Rheumatology (Oxford), 2016. **55**(10): p. 1786-90.
17. Cramp, F., et al., *Non-pharmacological interventions for fatigue in rheumatoid arthritis.* Cochrane Database Syst Rev, 2013(8): p. Cd008322.
18. Hewlett, S., et al., *Self-management of fatigue in rheumatoid arthritis: a randomised controlled trial of group cognitive-behavioural therapy.* Ann Rheum Dis, 2011. **70**(6): p. 1060-7.
19. Hewlett, S., et al., *Reducing arthritis fatigue impact: two-year randomised controlled trial of cognitive behavioural approaches by rheumatology teams (RAFT).* Ann Rheum Dis, 2019.
20. Ng, J.Y., et al., *Self-Determination Theory Applied to Health Contexts: A Meta-Analysis.* Perspect Psychol Sci, 2012. **7**(4): p. 325-40.
21. Bandura, A., *Social cognitive theory: an agentic perspective.* Annu Rev Psychol, 2001. **52**: p. 1-26.

For peer review only - http://bmjopen.bmj.com/site/about/guidelines.xhtml

22. White, C., in *Cognitive Behaviour Therapy for Chronic Medical Problems: A Guide to Assessment and Treatment in Practice.* 2001, Wiley: Chichester.
23. Craig, P., et al., *Developing and evaluating complex interventions: the new Medical Research Council guidance.* Int J Nurs Stud, 2013. **50**(5): p. 587-92.
24. Wood, S.J., et al., *Twelve tips to revitalise problem-based learning.* Med Teach, 2015. **37**(8): p. 723-729.
25. Nicklin, J., et al., *Measuring fatigue in rheumatoid arthritis: a cross-sectional study to evaluate the Bristol Rheumatoid Arthritis Fatigue Multi-Dimensional questionnaire, visual analog scales, and numerical rating scales.* Arthritis Care Res (Hoboken), 2010. **62**(11): p. 1559-68.
26. Gossec, L., et al., *Finalisation and validation of the rheumatoid arthritis impact of disease score, a patient-derived composite measure of impact of rheumatoid arthritis: a EULAR initiative.* Ann Rheum Dis, 2011. **70**(6): p. 935-42.
27. Pincus, T., et al., *Assessment of patient satisfaction in activities of daily living using a modified Stanford Health Assessment Questionnaire.* Arthritis Rheum, 1983. **26**(11): p. 1346-53.
28. Hewlett, S., et al., *Development and validation of a self-efficacy scale for use in British patients with rheumatoid arthritis (RASE).* Rheumatology (Oxford), 2001. **40**(11): p. 1221-30.
29. Dempster, M. and M. Donnelly, *Validity of the Perceived Health Competence Scale in a UK primary care setting.* Psychol Health Med, 2008. **13**(1): p. 123-7.
30. Williams, G.C., et al., *Autonomous regulation and long-term medication adherence in adult outpatients.* Health Psychol, 1998. **17**(3): p. 269-76.
31. Herdman, M., et al., *Development and preliminary testing of the new five-level version of EQ-5D (EQ-5D-5L).* Qual Life Res, 2011. **20**(10): p. 1727-36.
32. van Hout, B., et al., *Interim scoring for the EQ-5D-5L: mapping the EQ-5D-5L to EQ-5D-3L value sets.* Value Health, 2012. **15**(5): p. 708-15.
33. *National cost collection data*. Available from: https://www.england.nhs.uk/national-cost-collection/.
34. *Mileage allowances - Section 17* Available from: https://www.nhsemployers.org/pay-pensions-and-reward/agenda-for-change/nhs-terms-and-conditions-of-service-handbook/mileage-allowances/.
35. *Unit costs of health and social care 2019*. Available from: https://www.pssru.ac.uk/project-pages/unit-costs/unit-costs-2019/.
36. *BMA and Royal Pharmaceutical Society.* . 2019 ed. British National Formulary 76. London: BMJ Group and Pharmaceutical Press.
37. Dures, E.K., et al., *Reliability and sensitivity to change of the Bristol Rheumatoid Arthritis Fatigue scales.* Rheumatology (Oxford), 2013. **52**(10): p. 1832-9.
38. Bonfá, E., et al., *How COVID-19 is changing rheumatology clinical practice.* Nature Reviews Rheumatology, 2021. **17**(1): p. 11-15.
39. Sloan, M., et al., *The impact of the COVID-19 pandemic on the medical care and health-care behaviour of patients with lupus and other systemic autoimmune diseases: a mixed methods longitudinal study.* Rheumatology Advances in Practice, 2020. **5**(1).
40. Howren, A., et al., *Virtual rheumatology appointments during the COVID-19 pandemic: an international survey of perspectives of patients with rheumatic diseases.* Clinical Rheumatology, 2020. **39**(11): p. 3191-3193.
41. Kirby, T., *Rheumatologists rapidly adjust patient care during COVID-19 pandemic.* The Lancet Rheumatology, 2020. **2**(5): p. e258.
42. Haroon, M., et al., *Bristol rheumatoid arthritis fatigue scale is valid in patients with psoriatic arthritis and is associated with overall severe disease and higher comorbidities.* Clin Rheumatol, 2020. **39**(6): p. 1851-1858.

For peer review only

TABLE 1: OVERVIEW OF INTERVENTION STRUCTURE AND CONTENT

**Sessions 1-4**	**Key topics**	**Key handouts**
Engagement and validation	Identify fatigue drivers<br>Activity management	Fatigue overview<br>Activity diaries
Daily diary, goals, action planning	Boom and bust; avoidance and withdrawal<br>Drainers and energisers	Pacing<br>Goal setting<br>Activity diaries
Sleep and rest	Nature of sleep difficulties<br>Sleep myths and strategies	Sleep and relaxation<br>Activity diaries
Stress and relaxation	Symptoms of stress<br>Coping resources	Stress bucket<br>Activity diaries

For peer review only

For peer review only - http://bmjopen.bmj.com/site/about/guidelines.xhtml

TABLE 2: FREE-IA PARTICIPANT DEMOGRAPHICS

	Study Participants (n = 46)
**Sex (%)**	
Male	9 (19.6%)
Female	32 (69.6%)
Missing	5 (10.9%)
**Ethnicity (%)**	
White	39 (84.8%)
Black	1 (2.2%)
Prefer not to say	1 (2.2%)
Missing	5 (10.9%)
**Age in years (%)**	
< 40	5 (10.9%)
40 - 49	10 (21.7%)
50 - 59	15 (32.6%)
60 - 69	7 (15.2%)
70 - 79	3 (6.5%)
Missing	6 (13.0%)
**Site (%)**	
1 (South East England)	8 (17.4%)
2 (South East England)	7 (15.2%)
3 (South West England)	15 (32.6%)
4 (North West England)	10 (21.7%)
5 (South West England)	6 (13.0%)

For peer review only

For peer review only - http://bmjopen.bmj.com/site/about/guidelines.xhtml

TABLE 3: SUMMARY OF PARTICIPANT-REPORTED OUTCOME MEASURES AT ALL TIME POINTS AND MEAN DIFFERENCES WITH CORRESPONDING 95% CONFIDENCE INTERVALS

**Measure** (Scale range)	**T0** Mean (SD) (Range)	**T1** Mean (SD) (Range)	**T2** Mean (SD) (Range)	**T1-T0** Mean difference (95% CI)	**T2-T0** Mean difference (95% CI)
BRAF-NRS Fatigue Effect (0-10)	8.48 (1.19) (6.00 to10.00) (n=46)	6.68 (1.54) (4.00 to 9.00) (n=40)	6.03 (2.72) (0.00 to 10.00) (n=39)	-1.78 (-2.27 to -1.28) (n=40)	-2.41 (-3.29 to -1.53) (n=39)
BRAF-NRS Coping (0-10)	6.68 (2.25) (1.00 to 10.00) (n=41)	5.79 (2.53) (0.00 to 10.00) (n=34)	5.03 (2.72) (0.00 to 10.00) (n=34)	-0.59 (-1.53 to 0.34) (n=32)	-1.06 (-2.00 to -0.12) (n=32)
RAID Final Score (0-10)	6.40 (1.60) (1.87 to 9.25) (n=41)	5.57 (2.00) (1.65 to 8.79) (n=34)	5.54 (1.91) (1.30 to 8.79) (n=36)	-0.64 (-1.27 to -0.00) (n=32)	-0.61 (-1.32 to 0.10) (n=33)
BRAF-MDQ Physical Severity (0-22)	17.92 (2.82) (11.00 to 22.00) (n=41)	14.97 (4.16) (5.00 to 22.00) (n=34)	14.56 (5.22) (4.00 to 22.00) (n=34)	-2.44 (-3.75 to -1.12) (n=32)	-2.87 (-4.85- to -0.89) (n=30)
BRAF MDQ Living with Fatigue (0-21)	12.42 (4.95) (4.00 to 21.00) (n=41)	9.09 (6.10) (0.00 to 21.00) (n=34)	8.63 (5.88) (0.00 to 21.00) (n=34)	-2.75 (-4.52 to -0.98) (n=32)	-2.72 (-4.55 to -0.88) (n=30)
BRAF-MDQ Cognitive (0-15)	9.39 (3.93) (1.00 to 15.00) (n=41)	7.62 (3.82) (0.00 to 15.00) (n=34)	7.09 (3.51) (1.00 to 15.00) (n=34)	-1.84 (-3.19 to -0.50) (n=32)	-1.63 (-3.22, -0.05) (n=30)
BRAF-MDQ Emotional (0-12)	7.71 (3.16) (1.00 to 12.00) (n=41)	5.44 (3.51) (1.00 to 12.00) (n=34)	5.47 (3.52) (0.00 to 12.00) (n=34)	-1.47 (-2.51 to -0.42) (n=32)	-1.67 (-3.06 to -0.27) (n=30)
BRAF-MDQ Total (0-70)	47.43 (12.60) (21.00 to 66.00) (n=41)	37.12 (15.39) (14.00 to 68.00) (n=34)	35.75 (15.84) (9.00 to 66.00) (n=34)	-8.50 (-13.03 to -3.97) (n=32)	-8.88 (-15.00 to -2.77) (n=30)
MHAQ Mean Score (0-4)	0.84 (0.58) (0.00 to 2.38) (n=41)	0.72 (0.55) (0.00 to 2.13) (n=33)	0.81 (0.61) (0.00 to 2.00) (n=34)	-0.07 (-0.23 to 0.08) (n=31)	0.03 (-0.15 to 0.21) (n=31)
HCCQ (1-7)*	3.95 (1.50) (1.17 to 7.00) (n=39)	5.46 (1.36) (2.00 to 7.00) (n=34)	4.85 (1.69) (1.33 to 7.00) (n=36)	1.35 (0.65 to 2.05) (n=31)	1.01 (0.35 to 1.67) (n=32)
RASE (28-140)*	100.16 (12.20) (78.00 to 128.00) (n=38)	105.67 (13.36) (72.00 to 140.00) (n=33)	104.32 (16.21) (72.00 to 135.00) (n=35)	3.32 (-0.62 to 7.26) (n=31)	4.80 (1.00 to 8.60) (n=32)

SD, Standard deviation, 95% CI, 95% confidence intervals

** Higher scores indicate better outcome*

For peer review only - http://bmjopen.bmj.com/site/about/guidelines.xhtml

Table 4. Economic evaluation measures: resource use, costs and outcomes over six months of follow-up

**Resource use**	***n***	**Mean resource use per participant (SD)**	**Mean costs per participant (£)**	**95% CI**
A&E visits	35	0.14 (0.36)	23.71 (58.94)	
Outpatient visits	30	1.43 (1.76)	210.70 (258.05)	
Day cases	30	0.40 (1.33)	300.80 (999.20)	
Inpatient stays	30	0.10 (0.31)	224.57 (777.42)	
GP appointments	34	1.94 (2.37)	66.00 (80.69)	
Nurse appointments	34	1.56 (2.26)	16.91 (24.51)	
GP home visits	30	0 (0)	0.00 (0.00)	
Nurse home visits	30	0.07 (0.37)	1.47 (8.05)	
Medications	30	2.57 (1.41)	2729.66 (2796.45)	
Nurse helpline	35	0.66 (1.03)	37.13 (58.05)	
Carer contacts	35	5.94 (30.95)	68.34 (355.90)	
**Total cost (NHS/PSS perspective)**			3690.08 (3660.83)	2323.10 to 5057.05
Informal care contacts	35	71.33 (165.20)	621.99 (1440.58)	
Private healthcare			82.33 (180.38)	
Private carers			128.03 (365.83)	
**Total cost (patient perspective)**			624.83 (1072.68)	224.28 to 1025.37
**Outcomes**	***n***	**Mean QALYs**		
QALYs over the six month period	27	0.275 (0.105)		0.23 to 0.32

n, all available data were used for each type of resource use or outcome; SD, standard deviation; 95%CI, 95% confidence intervals

For peer review only

FREE-IA FLOW DIAGRAM OF PARTICIPANTS

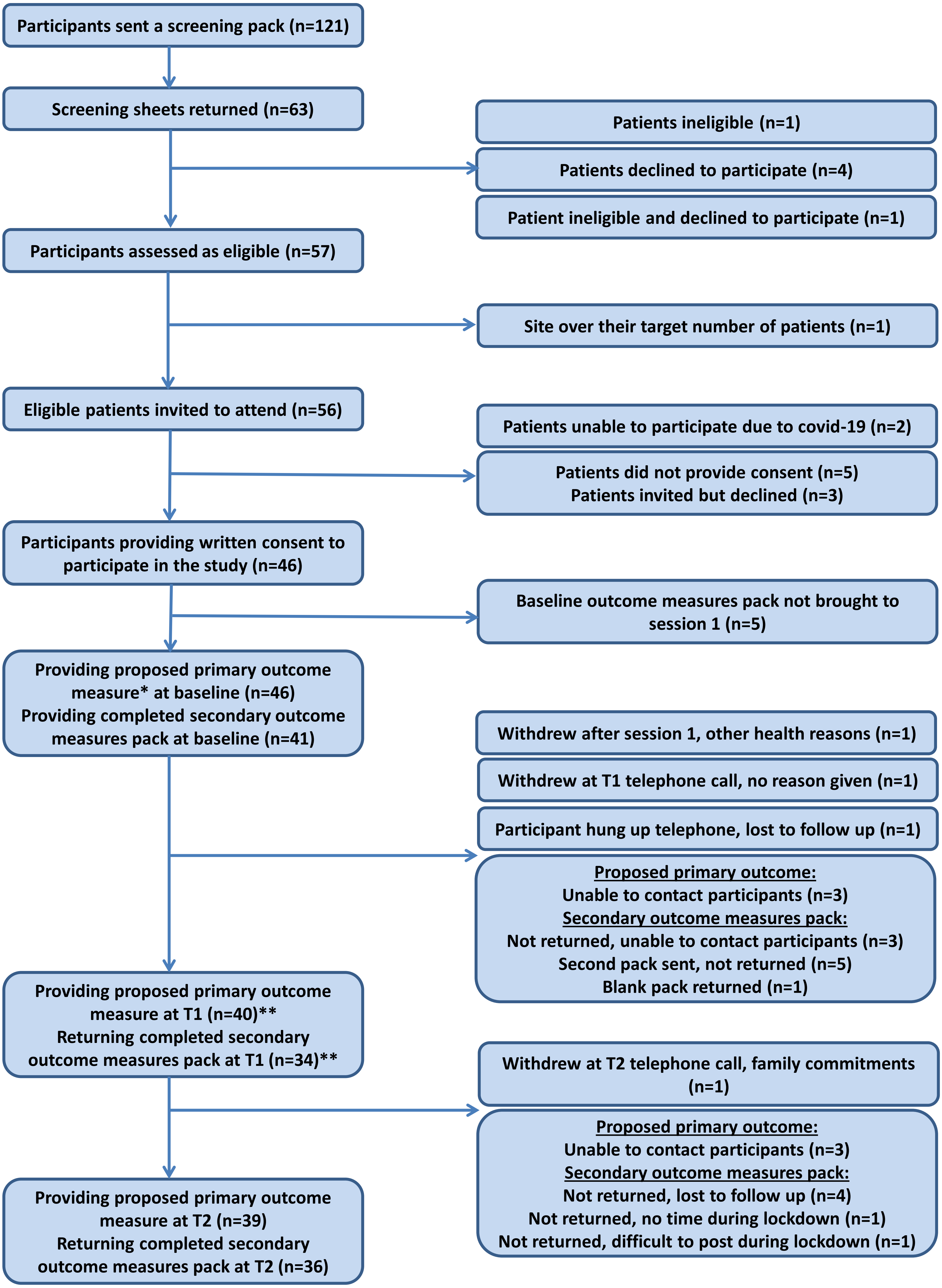

*The proposed primary outcome was collected by telephone

**Some participants did not return T1 outcomes but remained in the study and subsequently returned T2 outcomes

Fatigue: Reducing its Effects through individualized support Episodes in Inflammatory Arthritis (FREE-IA)

Summary of the intervention

The intervention is designed to provide rheumatology health professionals with a tool kit to support patients with an inflammatory arthritis (IA) to self-manage their fatigue impact. There are two parts to the intervention: health professional training and the intervention manual.

Training covers:

- Cognitive behavioural approaches (CBA)
- Experiences from a fatigue clinic and validating fatigue
- Identifying individual fatigue drivers and starting patient engagement
- Daily activity diaries
- Socratic or 'guided discovery' questions
- Boom and bust or withdrawal and avoidance
- SMART goal setting and practice with clinical vignettes
- Discussions of sleep, stress and the meaning of acceptance in IA

The manual provides the materials to deliver up to four sessions. Sessions 1 & 2 are core and Sessions 3 & 4 are optional.

- Session 1: Engagement and validation (establish that fatigue is an issue that the patient wishes to address with the health professional and engage them in taking some responsibility/action).
- Session 2: Review daily diary, goals, and action planning (review the patient's daily diary and reflect on whether this fits a boom/bust pattern of activity or a withdrawal and avoidance pattern of activity).
- Session 3: Sleep and rest (discuss potential factors contributing to disrupted or poor-quality sleep, forming helpful habits, and strategies to help the patient reduce their worry about sleep).
- Session 4: Stress and relaxation (support the patient to make connections between stressful circumstances, their fatigue, and how they cope with it, including the patient identifying their own symptoms of stress and recognising that stress comes from the interaction between circumstances (what's happening) and their response (thoughts and feelings and behaviours) to the circumstances).

At the end of each session, there is a checklist. At the back of the manual, there are session handouts for patients and tutor materials. There is homework and patients are asked to complete a daily activity diary between sessions and to try out changes to their activity and routine that they have discussed with the health professional.

The intervention is based on a cognitive-behavioural approach (CBA) using an 'Ask, don't tell' approach. CBA is based on the idea that symptoms do not shape how a patient responds to their IA, but rather the meaning that they make of those symptoms (e.g., that fatigue is unfair or a disaster). For this reason, health professionals see patients in clinic with similar symptoms/levels of disease, yet different ways of coping and different outcomes. To unpick this response, the patient needs to identify the links between their thoughts, feelings, and behaviours, and how these might be driving or exacerbating their fatigue. The health professional can help the patient to work out these links by asking questions to prompt the patient to reflect, including alternative ways of doing things. This can help the patient to generate their own ideas for ways forward and help ensure that the patient focuses on what is important to them. These are then translated into concrete goals for the patient to try.

For peer review only

Supplementary Table S1 (percentage of complete/imputed responses)

Measure	Time Point 0	Time Point 1	Time Point 2
BRAF-NRS Fatigue Effect (0-10)	100%	87.00%	84.78%
BRAF-NRS Coping (0-10)	89.13%	73.91%	73.91%
RAID Final Score (0-10)	89.13%	73.91%	73.91%
BRAF-MDQ Physical Severity (0-22)	89.13%	73.91%	73.91%
BRAF MDQ Living with Fatigue (0-21)	89.13%	73.91%	73.91%
BRAF-MDQ Cognitive (0-15)	89.13%	73.91%	73.91%
BRAF-MDQ Emotional (0-12)	89.13%	73.91%	73.91%
BRAF-MDQ Total (0-70)	89.13%	73.91%	73.91%
MHAQ Mean Score (0-4)	89.13%	71.74%	73.91%
HCCQ (1-7)	84.78%	73.91%	78.26%
RASE (28-140)	82.61%	71.74%	76.09%

For peer review only
